# Mobility and community at Mesolithic Lake Onega, Karelia, north-west Russia: insights from strontium isotope analysis

**DOI:** 10.1007/s12520-024-02129-8

**Published:** 2024-12-30

**Authors:** Rebekka Eckelmann, Laura Arppe, Alexey Tarasov, Łukasz Pospieszny, Lukáš Ackerman, Volker Heyd, Dmitry Gerasimov, Vyacheslav Moiseyev, Vanessa Fairbanks, Corrie Hyland, Kristiina Mannermaa

**Affiliations:** 1 Department of Cultures, Archaeology, University of Helsinki, Helsinki, Finland; 2 Finnish Museum of Natural History, University of Helsinki, Helsinki, Finland; 3 Institute of Linguistics, Literature and History, Karelian Research Centre, Russian Academy of Sciences, Petrozavodsk, Russia; 4 Institute of Archaeology, University of Gdańsk, Gdańsk, Poland; 5 Institute of Geology of the Czech Academy of Sciences, Prague, Czech Republic; 6 Peter the Great Museum of Anthropology and Ethnography (Kunstkamera), Russian Academy of Sciences, St. Petersburg, Russia; 7 Department of Anthropology and Archaeology, University of Bristol, Bristol, UK; 8 School of Earth Sciences, University of Bristol, Bristol, UK; 9 School of Archaeology, University of Oxford, Oxford, UK

**Keywords:** Mesolithic, Hunter-gatherer-fishers, Strontium, Mobility, Bioarchaeology, Geology

## Abstract

This study investigates the underlying mechanisms driving the formation of the largest known burial site of the Northern European Mesolithic, Yuzhniy Oleniy Ostrov (YOO). Radiogenic strontium isotope analysis (^87^Sr/^86^Sr) was used to identify group dynamics within the cemetery and examine the site’s place within local and supra-regional networks. The analysis of 57 humans and 31 animals was supported by the creation of an environmental baseline which identified four key geological zones and defines the strontium characteristics of Lake Onega. Only two individuals had strontium values indicating time spent outside of Lake Onega’s northern shores, where the majority of the burial population is likely to have resided. These results suggest that the YOO cemetery predominantly served as a burial place for those with semi-permanent residence in this area, with no significant evidence for gender-based relocation patterns indicative of patri- or matrilocal residency. However, materials seem to have travelled towards Lake Onega even over great distances and the presence of the two outlier individuals suggests these exchanges also involved long-distance travel of people. Our results align with an increasing number of studies that suggest a high degree of residential stability for Late Mesolithic foraging groups relying primarily on aquatic resources, yet simultaneously places the Lake Onega community within a wider Mesolithic communication network.

**Supplementary Information:**

The online version contains supplementary material available at 10.1007/s12520-024-02129-8.

## Introduction

As the largest known burial site of the Northern European Mesolithic, Yuzhniy Oleniy Ostrov^1^ (YOO) is one of the key sites for understanding human population dynamics and lifeways in the ancient past on the border between the Fennoscandic, Baltic and Central Russian cultural complexes of the Late Mesolithic and Early Neolithic.

In addition to the large number of burials, previous investigations also indicated a high diversity in some aspects of the site, such as craniometric traits, genotypes and burial find distribution (Gurina 1956; O’Shea and Zvelebil, 1984; Der Sarkissian et al. 2013; Zhirov 1940). As a result, the composition of the burial community (which here refers to the individuals interred and recovered at YOO) and its relation to the social function of the site have long been part of the research dialogue. In this respect, it has been suggested that the site may have been used as a common burial ground for multiple groups of Hunter-Gatherer-Fishers (HGF) with the island serving as a space for land-access negotiations, exchange of goods and reconnecting of community ties and mating networks (e.g. Jacobs 1995; Khlobistina 1978; Mannermaa et al. 2021; O’Shea and Zvelebil 1984; Schulting et al. 2022), although the scope of this network of interaction remains vague. This hypothesis was underscored by the presence of several burials with exceptionally numerous grave finds, including some with hundreds of animal tooth pendants and other artefacts of special interest, such as staffs depicting Eurasian elk (*Alces alces*) heads interpreted as power insignias and/or boat heads, as well as figurines in the shape of humans and snakes (Gurina 1956). A clarified chronology indicates a narrow time of usage for the site of ca. 200 years, coinciding with the 8.2 ka climatic cold event, potentially indicating a relationship between site usage and this phenomenon (Schulting et al. 2022).

All of these aspects make YOO a site of particular archaeological importance especially in the context of the wider Late Mesolithic in northern Europe. One way to investigate the underlying mechanisms that have led to the formation of the YOO site and local as well as supra-regional networks and group dynamics is radiogenic strontium isotope (^87^Sr/^86^Sr) analysis. As a geological proxy, ^87^Sr/^86^Sr analysis has been applied in archaeological research to separate local and non-local individuals, identify areas of origin for people, animals and materials, and investigate habitat ranges, in addition to a variety of socio-cultural questions regarding, for example gendered mobility patterns (e.g. Bentley 2006; Bentley et al. 2002; Boethius et al. 2022a, b; Ericson 1985; Montgomery 2010; Price et al. 2021).

Here, we applied ^87^Sr/^86^Sr and Sr concentration analysis on 57 humans and 31 animals, in order to examine whether the variability noted in other proxies (see above) would also be present in the ^87^Sr/^86^Sr signal of humans and fauna buried at YOO, potentially indicating a high variability in people’s (geographical) origins. Further, it was evaluated whether these values would allow us to estimate the geographical location of the resource catchment area of the buried individuals. To facilitate this, we also analysed 67 mostly modern environmental samples, to acquire information on the strontium baseline in the Lake Onega region. Combined, this could help to clarify the status of YOO as either as a localized, regional or cross-regional institution.

### The site

**Fig. 1:**
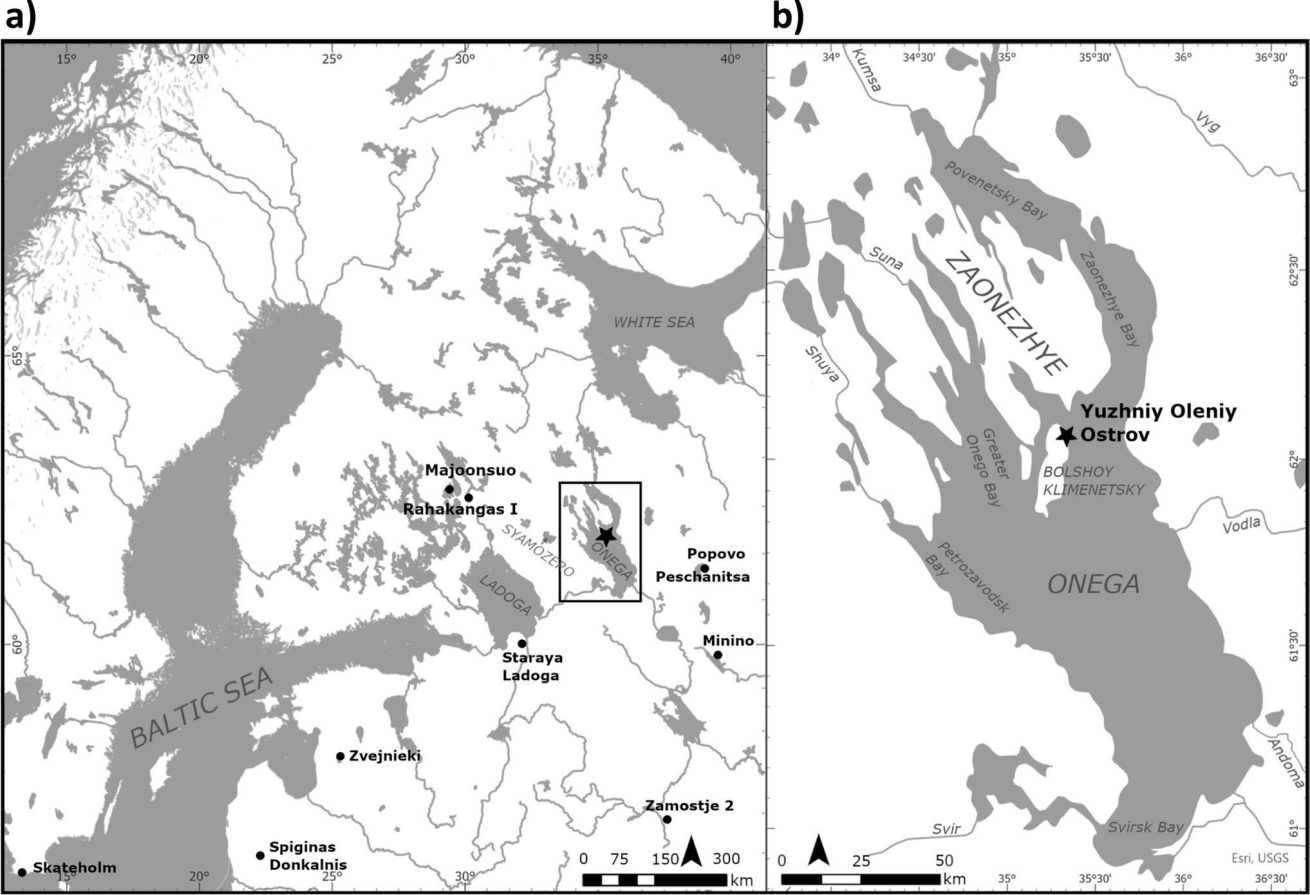
**a** Overview map showing the sites mentioned in the text; **b** Detail of the Lake Onega area with the location of the Yuzhniy Oleniy Ostrov burial site and relevant geographical points

The YOO burial site is located on the eponymous island in the northern part of Lake Onega in Russian Karelia (Fig. 1, 62°07′44.3″N and 35°34′33.9″E; Mannermaa et al. 2021) and was discovered during limestone quarrying operations (Gurina 1956). After reports of archaeological finds reached the local authorities, a team of the St. Petersburg Academy of Sciences under the leadership of Vladislav I. Ravdonikas and Nina N. Gurina excavated the site from 1936 to 1938, ultimately recovering 177 burials and more than 7000 artefacts (Gurina 1956; Ravdonikas 1956). Subsequent radiocarbon analyses have dated the burial activity at the site to a narrow time window of only ca. 200 years (centred at 8250–8000 cal BP) at the locally-defined end of the Mesolithic period (Schulting et al. 2022).

Even though an unknown number of burials had already been destroyed at the point of excavations due to the ongoing limestone mining operations,^2^ YOO remains the largest known Mesolithic burial site in northern Europe to this day. It is so far also the only securely identified Mesolithic (i.e. non-ceramics using hunter-gatherers) burial site in the wider Lake Onega area (Filatova 2004). The closest known other confirmed Mesolithic sites with human remains are Popovo (MNI = 10, Oshibkina 2016), Peschanitsa (MNI = 1–2, Oshibkina 2016), Rahakangas I (MNI = 1, Pesonen et al. 2014; Ahola et al. 2016) and Majoonsuo (MNI = 1, Kirkinen et al. 2022), all at a substantial distance (Fi.g 1). One aspect contributing to this situation is that, due to unique local geological conditions, YOO is one of the very rare Stone Age sites in Northern Fennoscandia and North-Western Russia where skeletal remains are preserved at all. The acidic nature of the local soils typically only rarely preserves organic materials in burials, which are usually identified as former inhumations only due to their form and the presence of characteristic ochre features (e.g. Ahola et al. 2016; Hakonen et al. 2023; Kirkinen et al. 2022). There are a small number of contexts from Lake Onega (less than 10 instances) and one from Lake Syamozero (ca. 60 km to the west of Lake Onega) which could potentially be counted among these red ochre graves and dated to the Mesolithic (Oshibkina 2008; Pankrushev 1978), but their identification remains uncertain (Filatova 2012).

This position of YOO as the only securely identified burial site in the area, the short time of occupancy combined with the extent of the site and the observed sizes of boreal HGF groups (e.g. Layton et al. 2012) strongly indicate that YOO served as a special and centralized cemetery for multiple resident communities (Gurina 1956; O’Shea and Zvelebil 1984; Schulting et al. 2022).

During the long period of time that YOO has been part of the research discourse, there have been various attempts to better characterize the site and its suggested special function as well as the people who created it. One aspect of this ongoing research has been the question of cultural and biological homo- or heterogeneity within the burial community, specifically after early osteological analyses led to the suggestion that some of the people at the site may have skeletal characteristics typical to populations from beyond the Urals (Yakimov 1960; Zhirov 1940). Even though that hypothesis has at this point become obsolete (Khartanovich 2006), the question of the geographical range of groups active in the site creation process has continued to be part of the research dialogue. During this time, analyses have found patterns of both a relatively high degree of variance ( e.g. in genetic and osteometric variability in the burial population; see Gokhman 1994; Der Sarkissian et al. 2013; Yakimov 1960) and strong coherence and similarity, for example in terms of mortuary practices and material culture. Both share indicators of cross-regional interaction (Gurina 1956; Mannermaa et al. 2019; O’Shea and Zvelebil 1984) and a firm rooting in the local material culture spanning the shores of Mesolithic Lake Onega and Lake Syamozero (Filatova 2004). V. Filatova (2004) proposed the term “Onega culture” (or “Onezhskaya culture”) for this group of sites, due to the shared characteristics of their settlement structures and material culture. While part of the wider Eastern Blade Technology complex (Sørensen et al. 2013; Tarasov 2018), the Onega culture as described by Filatova appears to be materially distinct from both the archaeological cultures of the White Sea and the Baltic Sea area. Instead, it’s closest cultural relatives (based on material culture) are the Butovo and Veretye cultures, east of Onega, which, however, are considerable older than YOO and represent the earliest occupation phases in the area (beginning around 9000 years cal BC) (Filatova 2004).

Of the over 300 Mesolithic sites currently known in the Lake Onega area, 80 have been excavated. The majority have not been radiocarbon dated but dated according to hypsometric position and typology (e.g. Tarasov 2018; Zobkov et al. 2019). They are found along the shores of the lake, often close to river estuaries, with multiple site concentrations (e.g. on the northern shore of Povenetsky Bay, on Bolshoy Klimenetsky Island, and Petrozavodsk Bay; see Fig. 7). The majority are interpreted as habitation sites, with 15 of the excavated sites containing partially subterranean dwelling structures that could have been in year-round use, as well as a smaller number of workshop sites (Filatova 2002, 2004; Tarasov pers. comm.).

In terms of location, Yuzhniy Oleniy Island lies to the east of the much larger Bolshoy Klimenetsky Island, from which it is separated by a water channel today measuring ca. 320 m, and has a modern surface area of ca. 2.7 × 0.5 to 0.7 km (Filatova 2002). Because continuous gravel mining has heavily altered the surface of the island, it is not possible to get a representative picture of what the area might have looked like in the Mesolithic. However, due to higher water levels at the end of the Boreal compared to today, the size of the island was likely substantially smaller and the distance to Bolshoy Klimenetsky bigger (Filimonova and Lavrova 2015; German et al. 2006).

Both islands are part of the Zaonezhye area, which here includes the peninsula of the same name, as well as the Sjar and Lizhma peninsulas and the Kizhi archipelago, altogether containing more than 300 islands and dominating the northern quarter of the Lake Onega area. Lake Onega itself is the second largest lake in Europe, with a water surface area of more than 9690 km^2^ and being fed by more than 1000 tributaries. It is part of the boreal forest (taiga) biome that today experiences humid, summer-warm climate, with mean temperatures varying from ca. − 10°C in January to c. 16°C in July (Filatov and Rukhovets 2012).

Recent palynological research from Razlomnoe Peat, Lake Onega (Krikunova et al. 2024) indicates that compared to today, winter temperatures during the 8.2 ka event were significantly colder, with low precipitation and thin snow cover, while summers were warm and comparatively dry. An intensification of forest fires is also reconstructed between 8300 and 8000 BP (Clear et al. 2014). It is hypothesized that Lake Onega’s size would have created its own milder micro-climate (Karpin 2014; Kondratyev et al. 1999) ameliorating the effects of the 8.2 ka event and leading to a potentially higher density of game animals, such as elk, close to the lake itself (Schulting et al. 2022). Combined with the theory that the depth of Lake Onega decreased the chances of fish mass winter kills due to hypoxia and the increased danger from forest fires, this would have made it a particularly attractive settlement area to human groups (Schulting et al. 2022).

### Principles of strontium isotope analysis

Radiogenic strontium isotope analysis (^87^Sr/^86^Sr) is employed in archaeology to investigate mobility and the potential provenance of humans, animals and other biological materials (e.g. Bentley 2006; Ericson 1985; Montgomery 2010). This is based on the principle that on a large scale, ^87^Sr/^86^Sr in the biosphere varies according to the lithology and age of local bedrock formations, and measured ^87^Sr/^86^Sr values are strongly related to the geographical origin of plants and secondary consumers. In general, present-day ^87^Sr/^86^Sr values reflect the initial ^87^Sr/^86^Sr composition and Rb/Sr of the source and the time elapsed since rock formation, because of the radioactive decay of ^87^Rb into ^87^Sr over time (e.g. Bataille et al. 2020). Accordingly, old felsic rocks with typically high Rb/Sr (e.g. granites) display the highest ^87^Sr/^86^Sr values (> 0.720), while young ferromagnesian (e.g. basaltic volcanic rocks) and carbonates show the lowest values (~ 0.703) due to their overall low Rb/Sr (Bataille et al. 2020; Peucker-Ehrenbrink and Fiske 2019). Topsoil predominantly inherits ^87^Sr/^86^Sr values from the underlying bedrock, but processes such as sea salt aerosols, the deposition of unconsolidated sediments like glaciogenic deposits (relevant to our study region), or different weathering processes in soil fractions can lead to differences between bedrock and soil cover (see Bataille et al. 2020).

Through dietary intake, the bioavailable strontium signal of the local foraging range enters the body of the consumer and is incorporated into body tissues. Due to its high density, level of mineralization and large crystal size archaeological tooth enamel is least likely to be affected by diagenetic changes (e.g. Hoppe et al. 2003). By investigating it for ^87^Sr/^86^Sr values, the obtained ratios can serve as a proxy for the region from which the diet was sourced during the formation of the enamel. By examining differences between local signals, variability in tissue ^87^Sr/^86^Sr values over time and comparisons within a sample population, it has been possible, for example, to detect immigrants from geological regions with a different ^87^Sr/^86^Sr signal in burial communities, identify sex-specific patterns of translocation and trace differences in habitat ranges (e.g. Bentley 2006; Montgomery 2010; Slovak and Paytan 2012).

However, several factors that limit the usability of ^87^Sr/^86^Sr results do exist. First, the strontium signal in body tissue is not a direct mirror of the geological background but depends on multiple factors, including the origin of the consumed food, which may not be sourced locally, and the concentration and absorbability of strontium in different ingested food sources (Lahtinen et al. 2021; Montgomery 2010; Plomp et al. 2020). Second, a relatively dense raster of background samples tied to local geology is needed to create a baseline for bioavailable strontium against which the archaeologically measured values are compared, especially in areas with complex geological histories (e.g. Bataille et al. 2020; Holt et al. 2021; Snoeck et al. 2020). In this respect, there are different approaches to collect bioavailable Sr baseline data, which include archaeological faunal and human samples, modern faunal samples preferably from animals with a small range, surface water, modern plant and/or soil leachate samples (ibid.). Yet, archaeological samples are not always available and have an unknown original range before deposition, whereas modern samples may differ from the values of the past, for example, due to anthropogenic influences (such as fertilizer or supplementation of lime in agricultural fields) that are capable of altering local ^87^Sr/^86^Sr signals. Currently, a joint strategy combining multiple sample types is recommended with modern environmental samples taken from areas not influenced by modern agriculture. The creation of a sufficiently representative raster is further complicated by the complexity of the local substrate and geology, as highly diverse formations necessitate denser sampling (e.g. Holt et al. 2021). In addition, statistical modelling of ^87^Sr/^86^Sr values depending on the local bedrock geology and other variables is increasingly applied to extrapolate values from baseline samples to cover larger areas (e.g. Bataille et al. 2020; Hoogewerff et al. 2019; Pouncett 2020). Its accuracy is, however, limited by similar factors as the creation of isoscapes from baseline samples only.

Sr elemental concentrations in archaeological research are often applied in support of dietary reconstructions based on the fact that the trophic level of consumers is negatively correlated with Sr concentrations as a result of biopurification processes along the food chain (Bentley 2006). They are also increasingly used in combination with ^87^Sr/^86^Sr, as Sr in food sources is also directly connected to consumer ^87^Sr/^86^Sr values and can help to clarify the origin of ^87^Sr/^86^Sr values in provenance studies (e.g. Boethius et al. 2024; Lahtinen et al. 2021). More rarely, Sr concentrations are utilized to ensure sample quality, by comparing measured Sr concentrations in archaeological materials with expected values based on modern comparisons (e.g. Budd et al. 2000). The paucity of reference values from different geological contexts and materials remains a severe limiting factor in all of these applications.

### Geology of the Lake Onega region

**Fig. 2:**
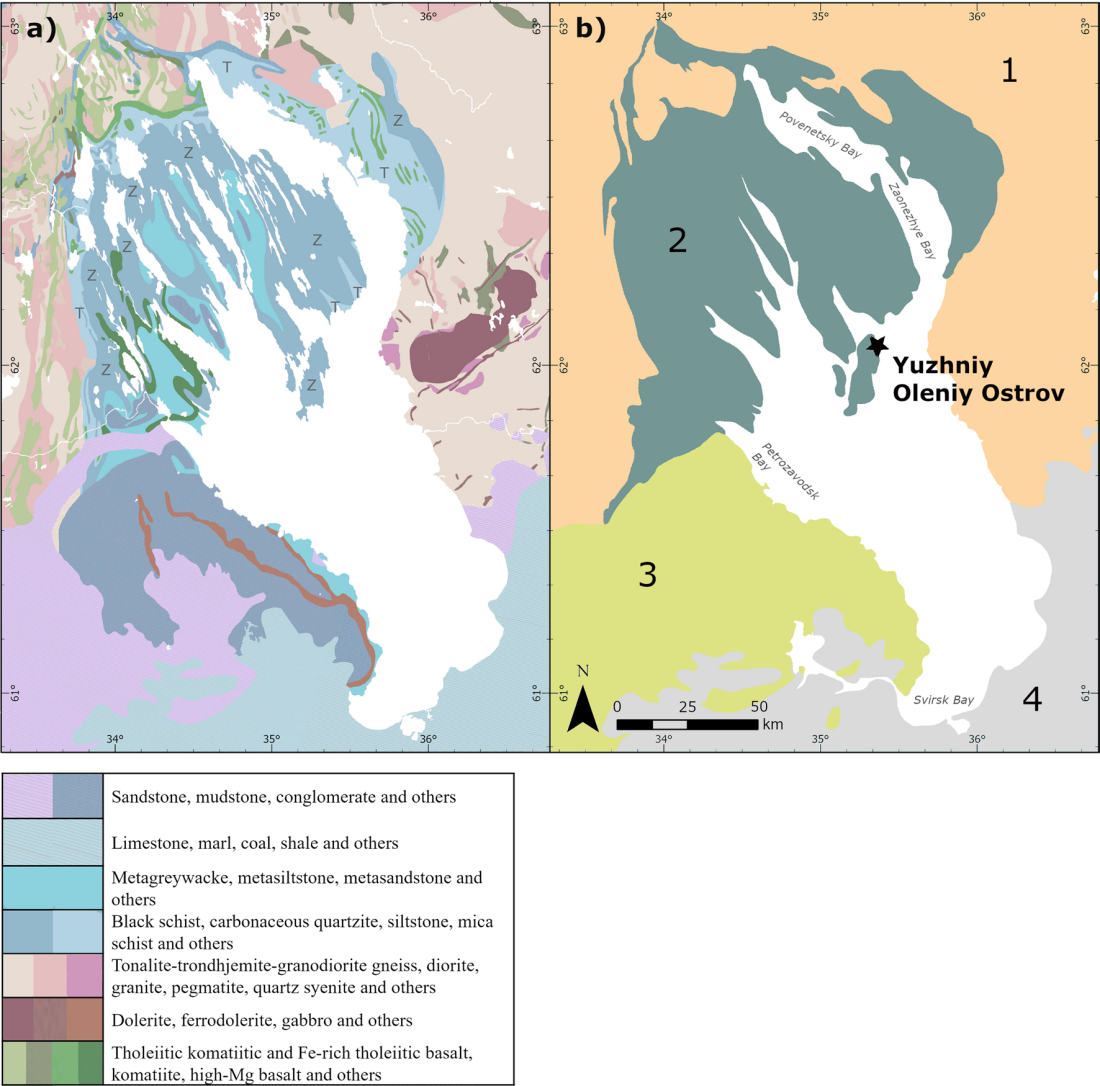
**a** Bedrock geology of the Lake Onega region (Koistinen et al. 2001, reproduced with the permission of The Federal Agency of Use of Mineral Resources of the Ministry of Natural Resources of the Russian Federation (MNRRF) from https://gtkdata.gtk.fi/fmd/ 9.11.2023). Z and T indicate the Zaonega Fm and Tulomozero Fm respectively. For a detailed version of the legend see SI 1 Fig. S1. **b** Simplified scheme (modified from Kolodyazhny et al. 2020; Kuznetsov et al. 2023) and four zones: 1. Hinterland - Mostly Archean rocks (3.2–2.65 Ga). 2. Northern shore zone - Mostly Palaeoproterozoic rocks (2.4–1.98 Ga). 3. South-western lake zone - Mostly Paleoproterozoic (ca. 1.9–1.75 Ga) and Vendian (650 Ma) sedimentary rocks. 4. EEP zone - Phanerozoic sedimentary rocks (509-298.9 MA)

Lake Onega formed after the retreat of the Fennoscandian Ice Sheet ca. 12,000–11,000 BP (Filatov and Rukhovets 2012). It is situated on two major geological provinces, with the northern and western parts of the lake sitting on the exposed Precambrian rock basement of the Fennoscandian Shield (FSS) and the south-eastern areas on the Eastern European Platform (EEP) covered by Vendian (650 Ma) and younger sedimentary rocks (e.g. Koistinen et al. 2001). The FSS, specifically the areas of Karelia and Kola, is comprised of some of the oldest and most complex bedrock formations in Europe (e.g. Nironen 2017). Based on prevalent rock types and bedrock age, four broad geological units can be divided in the Lake Onega area: (1) Archaean (~ 3.2 to 2.65 Ga) rocks dominated by the tonalite-trondhjemite-granodiorite (TTG) type and accompanied by various granitic rocks make up much of the eastern Lake Onega shoreline, and areas further inland to the north and west (Zone 1 in Fig. 2; “Hinterland”). (2) The northern shores and archipelago, including the Zaonezhye Peninsula and Yuzhniy Oleniy Island, consist of a highly complex Palaeoproterozoic (~ 2.4 to 1.98 Ga) low metamorphic grade sedimentary and volcanic succession, with organic-rich mudstones, greywackes and basalts of the Zaonega Formation being most common apart from the northern shoreline of Povenetsky and Zaonezhye Bay where dolostones, sandstones and mudstones of the Tulomozero Formation are dominant (Golubev et al. 2014; Melezhik et al. 2015) (Zone 2; “Northern Shore”). (3) The south-western shore is dominated by Paleoproterozoic (ca. 1.9 to 1.75 Ga) sandstone and quartzite of the Shoksha Formation (Kuznetsov et al. 2023) and terrigenous sedimentary rocks of Vendian (650 Ma) age (Kolodyazhny et al. 2020) (Zone 3; “South-Western Lake”). And (4) to the south and southeast of Lake Onega lie Devonian to Permian strata composed of mostly limestone, marl and terrigenous sedimentary rocks overlying the EEP (Zone 4; “EEP”).

Previous studies have demonstrated the extreme range of bedrock ^87^Sr/^86^Sr values on the FSS, resulting from its highly variable Rb/Sr ratios. The present-day modelled ^87^Sr/^86^Sr values of the Karelian bedrock province, making up much of north-eastern Finland and extending into Russian Karelia, including the study region around Lake Onega (corresponding to Zones 1 and 2), show median values at 0.7236 (IQR 0.0285) for the Archaean (Zone 1) and 0.7424 (IQR 0.1188) on the Proterozoic rocks (Kaislaniemi 2011 and references therein). Thus, while several other lithologies with likely low ^87^Sr/^86^Sr are present in the studied area (e.g. Melezhik et al. 2015), generally high ^87^Sr/^86^Sr values are likely to dominate Zones 1–2.

The preservation of osseous materials on Yuzhniy Oleniy Island itself is a result of this geological diversity, as the island is a rare outcropping of early Proterozoic Tulomozero Fm carbonate rocks in an archipelago otherwise dominated by sedimentary and volcanogenic rocks (Melezhik et al. 2015).

In contrast to the Precambrian rocks of the FSS in Zones 1–2, the area to the south and southeast of Onega (Zone 4) almost entirely consists of much younger, Phanerozoic carbonates and mixed sedimentary rocks of the EEP with relatively homogeneous Sr isotopic compositions spanning hundreds of kilometres (cf. Bataille et al. 2020; Petersone-Gordina et al. 2022; Piličiauskas et al. 2022; Price et al. 2020). Much of this Carboniferous to Permian cover consists of shallow marine carbonates, with expected ^87^Sr/^86^Sr values of ca. 0.7070–0.7090 based on the evolution of ^87^Sr/^86^Sr of the oceans (Popp et al. 1986; Veizer et al. 1999). While ^87^Sr/^86^Sr data for the intermixed terrigenous sedimentary rocks are not available, values modelled for the EEP by Bataille and colleagues (2020), remain consistently below 0.710. However, recent archaeological ^87^Sr/^86^Sr studies on the Devonian areas of the EEP in Estonia, Latvia, Lithuania, and Leningrad Oblast/Russia yielded noticeably higher bioavailable values than suggested by the model, at ca. 0.712 to 0.717 for human and animal enamel and plant samples (Oras et al. 2016; Petersone‐Gordina et al. 2022; Piličiauskas et al. 2022; Price et al. 2019, 2020). These higher values in comparison to those modelled or estimated above for the EEP carbonate bedrock most likely result from the Quaternary sedimentary cover.

Between these clearly contrasting bedrock domains are mixed terrigenous sedimentary rocks, mostly sandstones, siltstones, and conglomerates, of Proterozoic to Devonian origin (Zone 3). Their ^87^Sr/^86^Sr ratios are difficult to constrain a priori, as sedimentary rocks can display a wide range of Sr isotope values depending on their composition, but they are likely to be above those of the Phanerozoic carbonates of the EEP. The available information is limited, but apparently siliciclastic terrigenous sediments (mid to late Devonian; Kolodyazhny et al. 2020; Mikuláš et al. 2013) are also common in the very northernmost rim of Zone 4, close to the shores of Svirsk Bay. This area is thus likely to show more similarity to the ^87^Sr/^86^Sr levels of Zone 3 than those of the carbonate dominant EEP further south.

It follows, that the terrain beyond the immediate south-south-eastern shoreline of the lake located on the EEP should be clearly distinguishable from the northern areas of the FSS in terms of ^87^Sr/^86^Sr values, while differentiations within Zones 1–3 are likely to be more limited. Nevertheless, we still expect humans sourcing their diet primarily within one of the four described sub-regions (Zones 1–4) to be broadly distinguishable from each other, with the lowest values (≤ 0.717) expected to originate on the EEP.

Similarly, assuming the typical ^87^Sr/^86^Sr values for the Finnish and Archaean of the Karelian province (Kaislaniemi 2011) as being characteristic for Zones 1 and 2 around Lake Onega, we expect humans occupying the northern/north-western lake shores to yield the highest ^87^Sr/^86^Sr values among the studied specimens.

## Materials

### Bioavailable baseline samples

To establish a modern bioavailable Sr baseline, 34 plant samples, two water samples and eight faunal samples were acquired from areas in and surrounding Lake Onega during several field campaigns in 2017–2019. All samples were collected in non-urban areas and avoiding areas near roads and fields. The plant samples consisted of shrubs/herbs or tree leaf samples. At some locations, several types of plant samples representing different rooting depths were collected. All plant samples were collected at enough distance from the lake shore so as to not draw water from the lake. The modern faunal samples consisted of three fish from Lake Onega close to Yuzhniy Oleniy Island and one from Petrozavodsk Bay, two terrestrial voles (*Microtus *sp*.*) from Kizhi Island, one elk from Zaonezhye and one shell of a land snail from the YOO site. One water sample was collected directly from Lake Onega, close to Yuzhniy Oleniy Island, and one from a pond in the former quarry pit on the island.

Sampling for modern environmental baseline samples was conducted within the scope of archaeological field work expeditions. Accordingly, the selection of sampling localities was not systematically planned with respect to local geology but determined by the necessities of pre-determined field work schedules; additional sampling has so far not been possible. Therefore, the number of samples per zone varies, with 22 samples from the northern shore zone, nine from the south-western zone, four from the hinterland, four from the Phanerozoic rocks of the EEP in the immediate vicinity of the lake and five representing Lake Onega itself.

Due to the small number of samples available from the Onega vicinity in the EEP area, and the earlier discussed probability that the underlying rock type of the most proximal shoreline where the samples were obtained is not very representative of the EEP generally, the evaluation of Zone 4 was supplemented with samples we collected and measured from more distant areas (n = 9). These are mostly plants, but one sample is from an archaeological western capecaillie (*Tetrao urogallus)* bone from the Mesolithic-Neolithic site of Zamostje II/Moscow Oblast, Russia. While archaeological bone is generally not considered appropriate to evaluate in vivo ^87^Sr/^86^Sr values as it acquires the local soil ^87^Sr/^86^Sr signature due to diagenetic change, this change allows it to substitute as a proxy for the local bioavailable ^87^Sr/^86^Sr where no other samples are available (see Petersone-Gordina et al. 2022, Bentley et. al. 2004). In addition, literature values representing sites on the EEP were gathered and include data for eight samples from the Staraya Ladoga site in Leningrad Oblast/Russia (Price et al. 2019) and seven from multiple sites in Latvia (Petersone-Gordina et al. 2022). As a result, a total of 26 samples are available to represent the ^87^Sr/^86^Sr signal of the EEP. No literature data was available to supplement the hinterland area, but values from Archaean rocks of the same formation from Finland were considered as general guidelines (see Kaislaniemi 2011).

In addition to these samples, we report ^87^Sr/^86^Sr values for a further 14 modern environmental samples that were opportunistically collected from more distant areas, away from the four zones around Lake Onega, for example, from the shore of the White Sea (SI 1, Fig. S2). All baseline sample information, including the literature values, is listed in the supplementary information (SI 2, Table S1).

### Archaeological samples from YOO

All archaeological samples from YOO were collected from the dental tissues of humans and fauna from burials currently curated at the Anthropology and Archaeology sections of the Peter the Great Museum of Anthropology and Ethnography (MAE) in St. Petersburg (for details, see SI 2, Tables S2-S3). In total, 57 human individuals were sampled from YOO, providing 75 samples for ^87^Sr/^86^Sr analysis, 61 of which were also subjected to Sr concentration analysis. Almost all (71) samples were taken from tooth enamel, as per standard procedure to avoid contamination, but four teeth were also sampled for dentine to monitor diagenetic trends. In addition, 14 of the human individuals had enamel sampled twice on different teeth. Of the selected individuals, 29 were identified as female, 26 as male and two were not identified to sex. Biological sex for 28 females and 24 males was identified via DNA or peptide analysis and for two probable males and one probable female via osteological analysis (Batanina et al. in prep; Haak pers. comm.; Mittnik et al. 2018; Posth et al. 2023). All except for three individuals sampled were adults according to dental growth stages (Batanina et al. in prep; Gurina 1956), but as the enamel formation period for the majority of sampled teeth ends before the age of six years, the ^87^Sr/^86^Sr values reflect early childhood (AlQahtani et al. 2010). Preservation of teeth was bad overall; they appeared fragile, were of a greyish colour with occasional blackish spots, and showed visible cracks and flaked off enamel pieces.

The sampled faunal tooth enamel originated from 31 teeth (most of which are pendants) from twelve different graves. Of these, 25 represent Eurasian elk, four wild reindeer (*Rangifer tarandus*), one mountain hare (*Lepus timidus*) and one wild boar (*Sus scrofa*).

## Methods

### Strontium isotope and concentration analyses

The archaeological samples were analysed in the Bristol Isotope Group, School of Earth Sciences, University of Bristol in 2018 whereas the environmental samples were analysed at the Institute for Geology of the Czech Academy of Sciences in 2022.

Archaeological tooth specimens were rinsed by ultrasonication in ultrapure water (18.2 MΩ.cm). The process was repeated three times or more until the water in the vial remained clear, after which they were dried overnight at 80°C. Before cutting the sample, the surface of the sampling location on the tooth was cleaned with a stainless-steel grinding burr, to remove dirt and enamel discolouration. A wedge of enamel and dentine (ca. 0.5 mm wide, 1 mm deep) representing the complete growth axis of the enamel was removed using a flexible diamond-impregnated dental disk. A small piece of enamel (~ 20 mg), covering the area from the crown surface to the enamel-dentine junction, was separated and any dentine adhering to the enamel was removed. In some cases an additional piece of dentin (10–50 mg) was cut off. Chips of enamel and dentin were placed in micro-centrifuge tubes. The enamel and dentine fragments were transferred into glass vials, to a clean laboratory and again rinsed by ultrasonication in ultrapure water (18.2 MΩ.cm). The process was repeated three times, after which they were dried overnight at ~ 100°C. The dried enamel fragments were then powdered in an agate mortar and pestle under methanol to contain the powder. The methanol was evaporated under a heat lamp and the powder transferred to micro-centrifuge tubes.

Enamel powder and dentin fragments were weighed into perfluoroalkoxy (PFA) beakers and dissolved in a few millilitres of 7N HNO_3_ on a hotplate (120°C) overnight. The liquid was re-solidified on a hotplate (120 to 140°C), redissolved in ~ 300 µl of concentrated HNO_3_ and placed on a hotplate (140°C) overnight. The liquid was again re-solidified and redissolved in 3N HNO_3_. An aliquot of this solution, representing approximately 3 mg of solid enamel or dentine (containing ~ 100–300 ng of Sr) was taken for ion exchange chromatography. Strontium was separated from the enamel matrix using Sr Spec. resin (Eichrom 50 100 µm particle size; Horwitz et al. 1992). Approximately 100 µL of clean Sr Spec. was loaded on PFA micro columns and washed thoroughly with ultrapure water and 3 M HNO_3_. Dissolved enamel and dentine aliquots were then loaded in 500 µL 3N HNO_3_, the enamel matrix was eluted in 2 mL of 3N HNO_3_ and Sr was eluted with 1.5 mL of ultrapure water and collected in PFA beakers. Strontium fractions were dried on a hotplate (> 120°C), redissolved in ~ 300 µl of conc. HNO_3_ and 60 µl of 0.3 % H_3_PO_4_ and fluxed overnight on a hotplate (140°C) to break down any organics, and dried on a hotplate (120°C). High purity sub-boiling distilled acids were used throughout the process.

Strontium isotope ratios were measured on a Thermo-Finnigan Triton thermal ionisation mass spectrometer. Samples were loaded onto out-gassed rhenium filaments with a TaCl_5_ activator (Birck, 1986) and placed in the Triton source chamber. Amplifier gains and electronic baselines were measured at the beginning of each analytical session. Strontium isotopes were measured through a multi-dynamic ‘triple jump’ method (Thirlwall 1991) consisting of 20 blocks of 10 cycles with a 4.194 second integration time. The isobaric interference of ^87^Rb on ^87^Sr beams were corrected by monitoring ^85^Rb on each jump and assuming a natural ^85^Rb/^87^Rb ratio of 0.3857 adjusted for instrumental bass bias. Samples were corrected for instrumental mass bias by internal normalisation, assuming an exponential law and a fixed ^88^Sr/^86^Sr ratio of 8.375209 (Nier 1938; Russell et al. 1978). Long term reproducibility of ^87^Sr/^86^Sr for NIST SRM 987 processed through columns is 0.710248 ± 0.00001 (2σ, n = 113), whilst long term reproducibility of NIST SRM 1400 Bone Ash is 0.713118 ± 0.00001 (2σ, n = 53).

The modern plant leaf samples were powdered and decomposed in 60 ml Savillex digestion vessels using a mixture of 5 ml ultrapure 14 M HNO_3_ and 200 µl of 24 M HF acids for ~ 16 hours on a hot plate at 160°C. Subsequently, the solutions were dried and re-dissolved two to three times by 1 ml of 14 M HNO_3_ with the addition of 100–200 µl hydrogen peroxide to assure a complete decomposition. The snail shell and rodent enamel samples were decomposed in a 15 ml Savillex Teflon beaker using 2 ml of 14 M HNO_3_ for ~ 3 hours on a hot plate at 80°C and dried. Afterwards, all dried residues were dissolved in 1 ml of 1 M HNO_3_ and loaded on small columns (3 ml reservoir) filled with ~ 80 mg of a Sr resin (Triskem); Sr was isolated using the ion exchange chromatography protocol detailed in Pin et al. (2014). The ^87^Sr/^86^Sr isotopic analyses were carried out on W filaments in the presence of a Ta activator (Charlier et al. 2006) using a Thermo Triton Plus thermal ionization mass spectrometer (TIMS) operated in a static mode. During the course of this study, the NIST SRM 987 yielded a ^87^Sr/^86^Sr of 0.710251 ± 0.000006 (2σ, n = 5).

All sampled archaeological enamels as well as dried muscle tissue from three modern fish were also subjected to Sr concentration analysis. The former was primarily to ensure sample integrity, the latter to assess the local aquatic availability of strontium. Archaeological tooth enamel Sr concentrations were measured by ICPMS (Lewis et al. 2017). Fish samples were manually separated from bone, cleaned in a sonicator unit, minced, and dried at 60°C for 24 hours. Subsequently elemental composition was determined by ALS Scandinavia AB Luleå using inductively coupled plasma mass spectrometry (ICP-MS) according to SS-EN ISO 17294-2:2016, US EPA Method 200.8:1994 with prior sample nitric acid/hydroperoxide digestion with a trace of HF in a microwave oven (SS-EN 13805:2014) and a measurement precision of ≤ 7.5%.

### Data analysis

Outliers were examined via Tukey’s method and removed from further statistical tests to not violate test parameters but not excluded from overall data evaluation (see Results). After removal of outliers, data normality was verified using the Shapiro-Wilk test and equal variance via an *F*-test. The non-parametric Mann-Whitney *U*-test was employed to identify differences in median values between biological sexes and spatial clusters. The significance level accepted for statistical tests was p < 0.05 and the test is considered appropriate for the inclusion of groups with very differing sample sizes (e.g. Zimmerman 1987). A Kruskal-Wallis test for equal medians combined with Dunn’s post-hoc test was applied to investigate differences between the samples originating from the four different geological zones. The programs RStudio, Past4 and Microsoft Excel were used to analyse this data.

Statistical tests related to biological sex were calculated only for the individuals with secure sex identifications via DNA or peptide analysis. Other individuals were excluded from further statistical tests.

Spatial groups were identified according to visible breaks in the distribution of burials and refer to the northern and southern clusters of burials at the site, separated by ca. 20 m of undisturbed soil containing no burials. This separation was distinct enough to be already established during the excavations and has been maintained in research since (e.g. Gurina 1956; Jacobs 1995; O’Shea and Zvelebil 1984). Separation into southern, northern and central clusters could also be possible (Gurina, 1956), but due to the damage caused by the mining operation spatial delimiters are difficult to securely establish. For the purpose of this paper the established framework consigning burials 1–46 to the southern cluster (n = 6) and 47–170 to the northern cluster (n = 58) will be used (e.g. Gurina 1956; Jacobs 1995; O’Shea and Zvelebil 1984). There are indications that sample 5776-40 likely derived from burial 2, but since this assignation is not completely secure the sample was excluded from statistical evaluation.

## Results

### Strontium concentrations

The archaeological samples exhibited a wide range in Sr concentrations, with human values extending from 23 to 283 ppm and archaeological animals from 103 to 1697 ppm (SI 1, Fig. S3). While the former fall into the known range of human samples from uncontaminated sources (e.g. Plomp et al. 2020; Sowden and Stitch 1957), the highest values within the animal sample are of concern. Even though they all derive from elk, a herbivore whose Sr concentrations are expected to be higher than those of most omnivores or carnivores (Schoeninger 1985), values above 1000 ppm are so far unknown from previous studies, where herbivores usually yielded Sr contents below 500 ppm (e.g. Britton et al. 2009; Evans et al. 2007; Groot et al. 2020). There is no spatial pattern to the distribution of these samples at the site and at this point we do not have an explanation for the considerable elevation of Sr content in these samples, but possible causes may include contamination, severe diagenetic effects or analytical issues. Because of these potential concerns, combined with the majority of animal values falling within a more acceptable range for herbivores, we excluded the group with very high concentration values (> 1000 ppm; n = 10), from analysis, leaving 15 elk samples.

Nevertheless, the ^87^Sr/^86^Sr isotope ratios of samples with [Sr] > 1000 ppm (mean ^87^Sr/^86^Sr=0.7333 ± 0.0042; mean [Sr] = 1337 ppm ± 238) were broadly similar to those detected in the elk samples with more typical strontium concentrations (mean ^87^Sr/^86^Sr=0.7310 ± 0.0025; mean [Sr] = 337 ppm ± 160). Interestingly, they did not show any tendency towards the rather low (see below) ^87^Sr/^86^Sr signature of the burial site, as would be expected from contamination. However, a further exploration of the anomalous concentrations is outside the scope of this study.

The Mann-Whitney *U*-test did not identify statistically significant differences between Sr concentrations and biological sex or site cluster.

The fish tissue samples yielded Sr contents from 9.7 to 24 ppm, with the lowest value stemming from a zander (*Sander lucioperca*) and the higher values from two European perches (*Perca fluviatilis*) (SI 2, Table S4).

### ^87^Sr/^86^Sr values of modern baseline samples

As anticipated, the modern environmental samples collected to characterize the bioavailable ^87^Sr/^86^Sr levels around Lake Onega showed high variability in isotopic values. The ^87^Sr/^86^Sr values of the 34 plant samples varied between 0.7112 and 0.7500 (mean 0.7227 ± 0.0074, median 0.7217), four samples from terrestrial fauna yielded values from 0.7096 to 0.7323 (mean 0.7237 ± 0.0101, median 0.7264) and four fish ranged from 0.7225 to 0.7262 (mean 0.7236 ± 0.0017, median 0.7228), altogether higher than the Lake Onega water, which had a ^87^Sr/^86^Sr value of 0.7202. Plants collected at the same spot had similar values and did not exhibit differences in ^87^Sr/^86^Sr related to rooting depth.

**Fig. 3:**
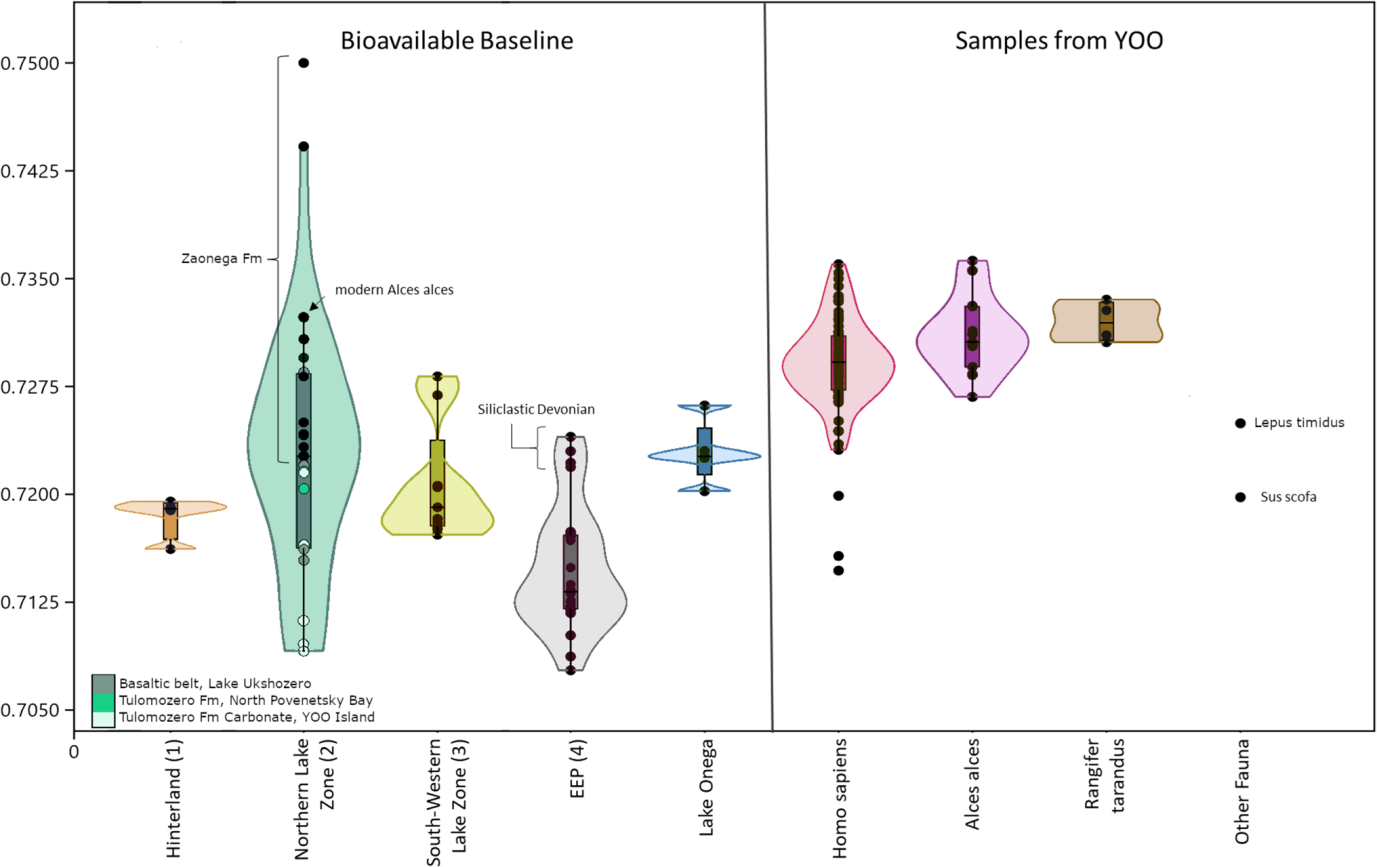
Overview of ^87^Sr/^86^Sr values used to characterize the four geological zones of the research area and Lake Onega itself, as well as the archaeological human and faunal enamel samples from YOO (SI 2, Table S1-S3). The EEP zone includes literature values (Petersone-Gordina et al. 2022; Price et al. 2019). The single sampled modern elk from the Zaonezhye peninsula (see arrow) is argued to be the best analogy of ^87^Sr/^86^Sr values in humans as well as other large vertebrates inhabiting the northern lake zone. The values derived from Tulomozero Fm carbonates, basaltic rocks and the siliclastic Devonian rocks are not representative of the typical bedrock within their zones. See “^87^Sr/^86^Sr values of modern baseline samples” and “Local geology and sources of strontium” sections for further discussion

In general, the expectations regarding the proposed four bedrock zones were fulfilled (Table 1, Fig. 3), with the EEP (Zone 4) yielding the lowest results, intermediate values in Zones 1 and 3 and the highest in Zone 2, especially in the area of the Zaonega formation. These trends hold, even though the statistical tests identified significant differences only between the low values of Zone 4 and the high value Zones 2 (p = 5.357e-5) and 3 (p = 0.0072). We additionally note, that within Zone 3 (^87^Sr/^86^Sr 0.717–0.728), the paleoproterozoic sedimentary lithologies closer to the shore (Fig. 2a) show similar ^87^Sr/^86^Sr values (0.717–0.727) to those of Vendian age (0.718–0.728) further west (Fig. 2a), thus offering no additional geographic resolution.

**Table 1:** Summary statistics of the ^87^Sr/^86^Sr values of the baseline samples used to describe the four different geological zones

	Zone 1: Hinterland	Zone 2: Northern Lake	Zone 3: South-Western Lake	Zone 4: EEP
N	4	22	9	26
Min	0.7162	0.7091	0.7172	0.7078
Max	0.7195	0.7500	0.7282	0.7240
Mean	0.7184	0.7240	0.7207	0.7148
Stand. dev	0.0015	0.0100	0.0041	0.0043
Median	0.7190	0.7230	0.7191	0.7132

The EEP zone includes literature values (Price et al. 2019; Peterson-Gordina et al. 2022; SI 2. Table S1)

Evaluating in more detail the lithologies represented by the baseline samples in the northern lake zone (2), we observe that the rock types common to the Zaonega formation, which makes up most of the land territory of the northern shore area and archipelago, are severely underrepresented by the sampling: out of the 22 samples for Zone 2, only 11 originate from the Zaonega Fm. They are concentrated on the higher end of the ^87^Sr/^86^Sr value range for Zone 2 (Fig. 3, black symbols in Zone 2), varying from 0.7227 to 0.7500 (mean 0.7304, median 0.7282). At the same time, the Tulomozero Fm, with its main continuous occurrence to the north of Povenetsky Bay and Zaonezhye Bay, is represented by a disproportional seven samples (due to the many samples collected on the Yuzhniy Oleniy Island). These stood out as predicted, showing the lowest values of Zone 2. The samples in question, a land snail shell and quarry pond water, were collected on exposed rock of the Tulomozero Fm carbonate formation yielding low ^87^Sr/^86^Sr values of ca. 0.709, while shrub leaves yielded values of 0.7112–0.7216. Even if the snail value should be treated with some caution, due to the minimal range of snail movement, adding these to the two samples collected to the north of Povenetsky Bay, the ^87^Sr/^86^Sr values of the samples on Tulomozero Fm range from 0.709 to 0.7220. Additionally, the remaining four samples with values from 0.7154 to 0.7285 (mean 0.7207, median 0.7194) represent basaltic rocks, also a minor occurrence in Zone 2 (green areas in Fig. 2a). Thus, the majority of baseline samples for Zone 2 in fact derive from substrata that are not representative of the most common bedrock formation (Zaonega Fm) in the area.

### ^87^Sr/^86^Sr values of the archaeological samples from YOO

The archaeological faunal samples yielded ^87^Sr/^86^Sr values from 0.7249 to 0.7365 (mean 0.7311 ± 0.0027, median 0.7309) while the one sampled boar tooth from burial 114 (acc. no. 5716 − 527, specimen 47000) was identified as an outlier at 0.7198 (Fig. 3).

**Fig. 4:**
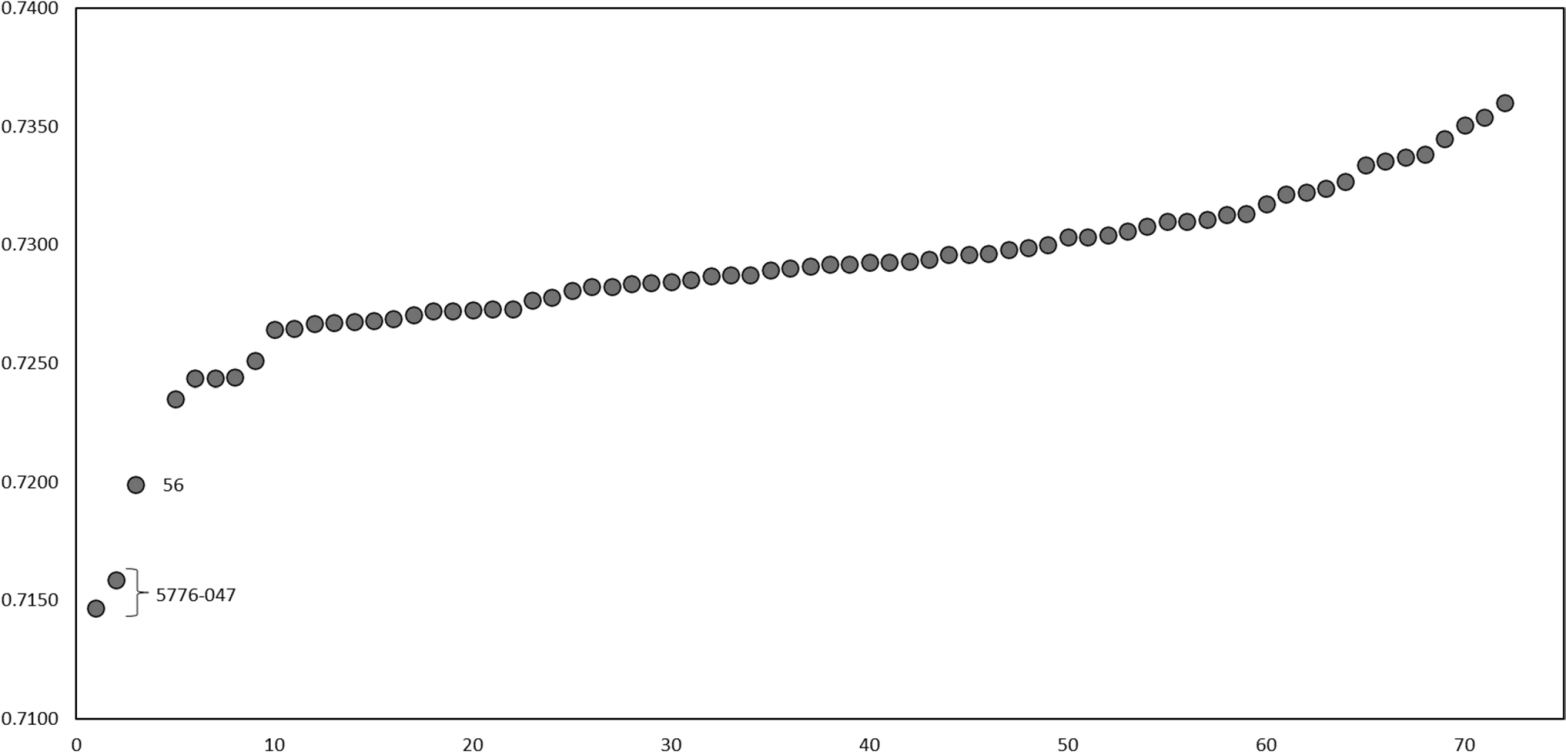
Human enamel ^87^Sr/^86^Sr values at Yuzhniy Oleniy Ostrov. Values are in ascending order. The three lowest values presented with their identifiers are statistical outliers. The lower two originate from the same individual (5776-047)

The ^87^Sr/^86^Sr values of the archaeological human enamel varied between 0.7235 and 0.7360 (mean 0.7293 ± 0.0027, median 0.7292) (Fig. 4). Three outliers were identified, two of which originate from individual 5776-047 with no known burial number at 0.7147 and 0.7158 and one of two samples from individual 56 (5776-007) at 0.7199, both with values falling far below those of the other samples. These outliers were removed from subsequent statistical analysis to maintain test parameters and both Shapiro-Wilk (*p* = 0.4856) and *F*-test (*p* = 0.8061) were passed after their removal. They were still considered in the overall evaluation though and a trial of including them into the tests did not change the significance of results.

The four human dentine samples yielded values (0.7195–0.7280, mean 0.7223 ± 0.0038, median 0.7210) that are lower than the corresponding enamel, consistent with the expected direction of diagenetic change towards the low values of the Tulomozero Fm carbonate bedrock of the burial site.

**Fig. 5:**
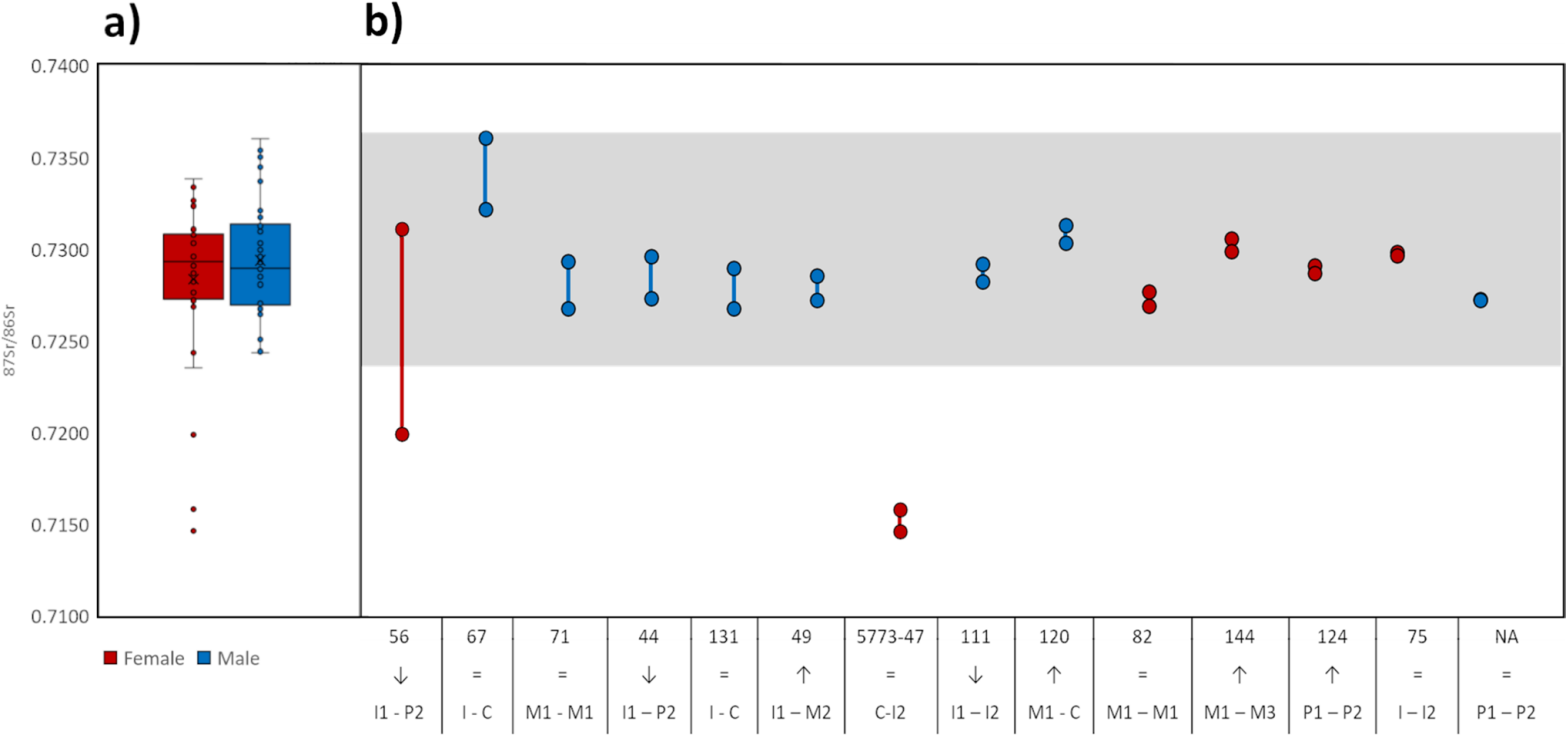
**a **Human enamel ^87^Sr/^86^Sr values at Yuzhniy Oleniy Ostrov according to sex (female n = 25, male n = 31), **b** ^87^Sr/^86^Sr values in double-sampled individuals sorted according to amplitude, with sex indicated by colour, as in panel (**a**). In (**b**), numbers below the horizontal axis indicate burial number, arrows indicate the direction of change in values over time based on the order of tooth mineralization and the lowest row indicates the analysed tooth types. The equal sign (=) indicates pairs of teeth with the same or very similar tooth mineralization ages. The gray zone indicates the distribution of all human non-outlier values from YOO

**Fig. 6:**
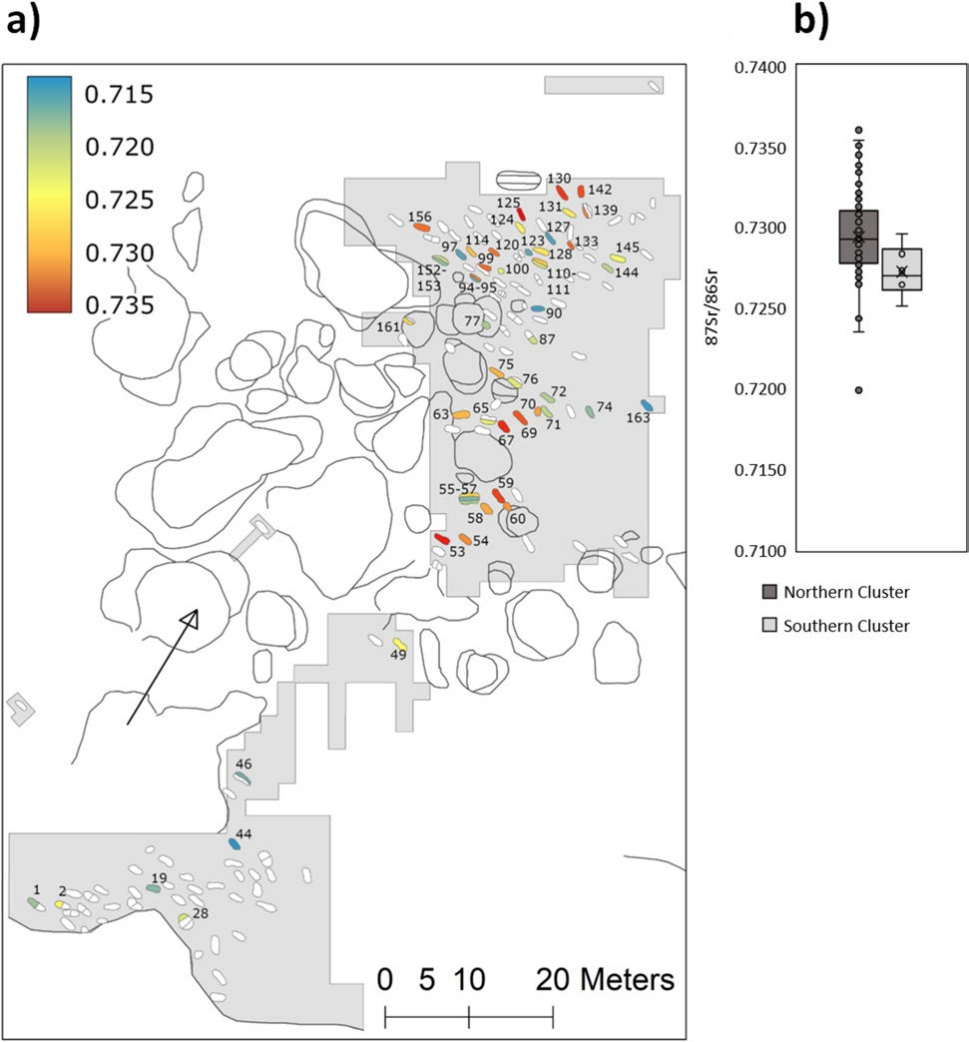
**a** Spatial distribution of human ^87^Sr/^86^Sr values at the Yuzhniy Oleniy Ostrov burial site. **b** Box plot illustrating the higher variance and higher ^87^Sr/^86^Sr values detected in samples from the northern cluster

Thirteen individuals for whom two enamel samples from different teeth were obtained yielded variation in ^87^Sr/^86^Sr values with a magnitude of 0.0001 to 0.0039 (mean 0.0014 ± 0.0011, median 0.0010). An additional outlier (burial 56, 5776-007) showed a much wider intra-individual difference of 0.0112 (Fig. 5). Differences in ^87^Sr/^86^Sr values according to sex were not significant in the overall sample (Mann-Whitney *U*-test *p* = 0.2132, Fig. 5) and neither was there a significant difference in intra-individual variability of values between male and female individuals with two samples (Mann-Whitney *U* test *p* = 0.0673; 56 as an outlier was not included). Spatially, there was a significant difference (Mann-Whitney *U*-test p = 0.0233) between the individuals in the northern (mean 0.7296 ± 0.0003, median 0.7293) and the southern (mean 0.7272 ± 0.0006, median 0.7293) clusters of the cemetery, with the southern area showing overall a narrower distribution with a noted absence of ^87^Sr/^86^Sr values above 0.7300 (Fig. 6). Even though the used statistical tests account for differences in group sizes, it should still be kept in mind that these differences are very big in the current case and the results should be treated with caution. The map of the site showing the spatial distribution of samples (Fig. 6) possibly also points towards less distinct groupings of similar values (e.g. an assemblage of higher ^87^Sr/^86^Sr value burials in the south-western edge of the northern cluster) but these remain obscure.

## Discussion

### Local geology and sources of strontium

As predicted, the data provided by biological baseline samples exhibited a very high variance in ^87^Sr/^86^Sr values, reflecting the complex makeup of the bedrock in the study area. The limitations in the number and representativeness of baseline samples, combined with the high variability of the geological background, did not allow for the creation of a high resolution bioavailable ^87^Sr/^86^Sr isoscape for the wider Lake Onega area.

**Fig. 7:**
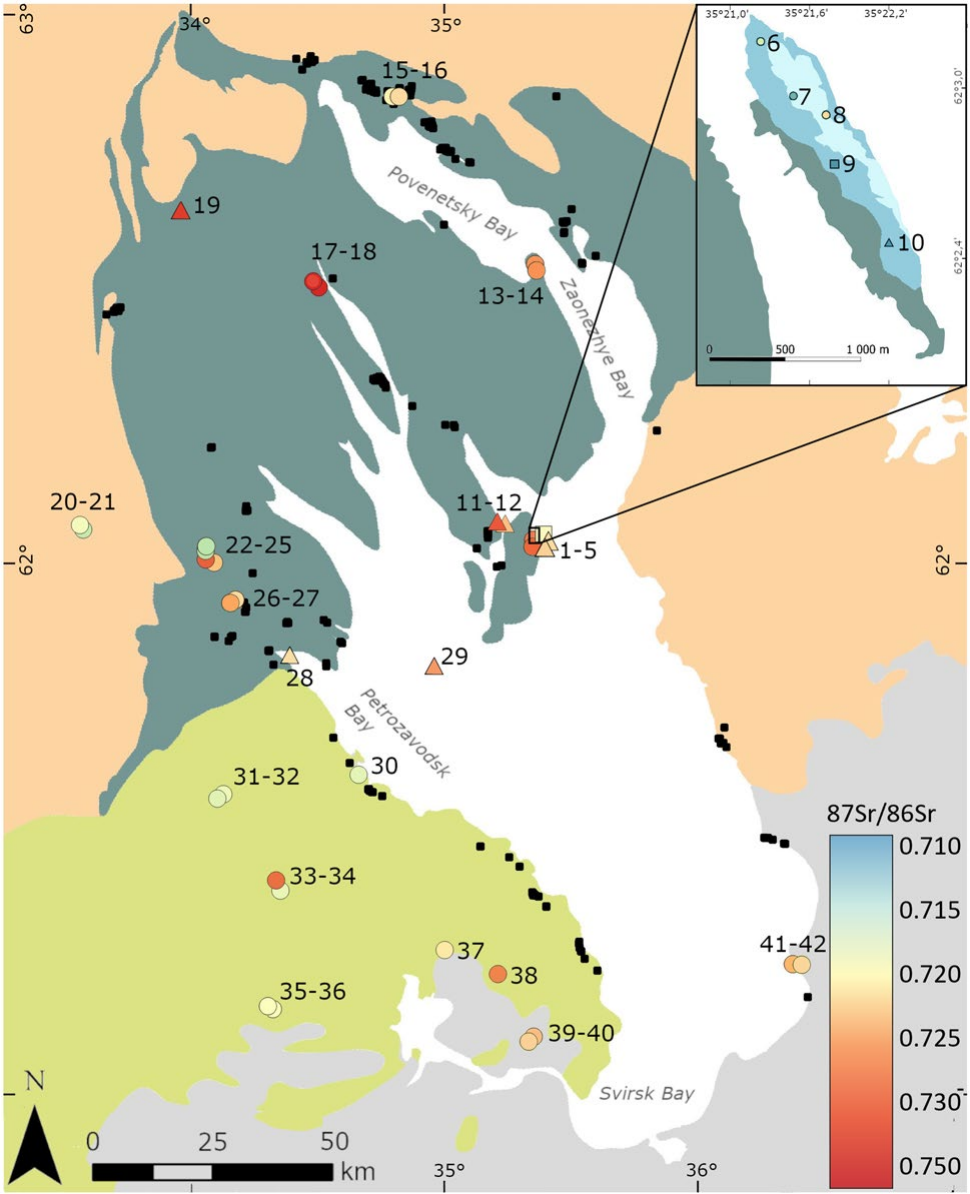
Lake Onega area and detail of Yuzhniy Oleniy Island with ^87^Sr/^86^Sr values of bioavailable baseline samples (color-coded; circles represent plants, squares water and triangles faunal samples; for numbered list see SI 2 Table S1) and the location of sites currently identified as Mesolithic (black squares; Filatova 2004, Tarasov, pers. comm.). The blue areas indicated on Yuzhniy Oleniy Island are Tulomozero Fm carbonate outcrops (drawn according to Golubev et al. 2014, Fig. 4)

Yet, the predicted larger scale trends for the four geological zones were corroborated, while there was substantial overlap between zones as well (see Fig. 3, 7).

The hinterland zone (1), though only represented by a small number of samples, yielded a relatively narrow and low range of ^87^Sr/^86^Sr values. This conforms with previous assessments by Kaislaniemi (2011) regarding the distribution of ^87^Sr/^86^Sr values on Archaean units of the Karelian bedrock province in Finland, whose continuation across the border constitutes the majority of our hinterland zone. The TTG rocks dominant in Zone 1 are characteristically low in potassium which commonly leads to relatively low Rb contents, as Rb usually replaces potassium in the crystal structure of minerals like K-feldspars and micas. Kaislaniemi (2011) observed that the Proterozoic Karelian formations in Finland have a much higher variance and higher ^87^Sr/^86^Sr values compared to the Archaean province, linked to their generally higher Rb/Sr ratios resulting in higher ingrowth of radiogenic ^87^Sr. This is mirrored in Proterozoic Zone 2, which displayed an extensive range and the highest overall ^87^Sr/^86^Sr values. Many of the most common rock types of Zone 2 belonging to the Zaonega Fm (mudstones and shales particularly), can display very high Rb concentrations (Reeder et al. 2005), giving rise to high ^87^Sr/^86^Sr values with age. At the same time, the relatively small occurrences of various basaltic rocks, as well as the presence of carbonate rocks, mainly of the Tulomozero Fm, extend the range down to values as low as 0.709. South of Povenetsky Bay, the Tulomozero Fm carbonates are a highly localized phenomenon rather than a large scale signature applicable to the wider Zone 2 area (see Golubev et al. 2014; Makarikhin et al. 2006). However, on the northern side of Povenetsky Bay and Zaonezhye Bay, the carbonates contained in the Tulomozero formation (Fig. 2a) may potentially lead to lower ^87^Sr/^86^Sr ratios being more common in that area.

Accordingly, our results for Zone 2 are not truly representative of the ^87^Sr/^86^Sr values most common to Zone 2, as the Zaonega Fm, which covers the majority of the terrain south of Povenetsky Bay, is strongly underrepresented in favour of more localized phenomena. Consequently, the samples derived only from the Zaonega Fm are more representative of Zone 2 as a whole and, overall, substantially higher than the mean ^87^Sr/^86^Sr calculated from all samples in the zone (Fig. 3). This is corroborated by one of the highest values (0.732), which was produced from a modern elk incisor. As animals moving through space represent an averaged ^87^Sr/^86^Sr signal of their habitat, they are a better indicator of the ^87^Sr/^86^Sr area signal than plants, which only indicate the spot of their growing depending on rooting depth (e.g. Bentley, 2006; Maurer et al. 2012). This is especially true in highly complex geological backgrounds (see Hoogewerff et al. 2019; Snoeck et al. 2020). While elk ranges are highly dependant on habitat conditions and differ significantly between populations, elk from Finland living in similar conditions to the elk analysed here have reported habitat ranges of 15–40 km^2^ (Suomen hirvikannan hoitosuunnitelma 2014) and mean migration distance at a latitude of 58–63° is ca. 8–28 km on average (Singh et al. 2012). As the size of the zonation we are applying well exceeds this habitat range by order of magnitude, we consider the elk incisor a good representation of the average terrestrial ^87^Sr/^86^Sr in the northern archipelago zone. Accordingly, its high value is another indicator that the average local ^87^Sr/^86^Sr is higher than our baseline indicates, which also means that the gap in values between 0.744 and 0.750 is likely an artefact of our sampling strategy.

It follows that while there is still a substantial overlap between the ^87^Sr/^86^Sr values recorded for the south-south-western shore zone (3) and the northern zone (Fig. 3), this overlap is drastically reduced if only the Zaonega Fm values are considered. The overlap is predominantly a result of the overrepresentation of the Tulomozero Fm and basaltic areas in the Zone 2 samples. Excluding those, Zone 3 is characterized by considerably lower ^87^Sr/^86^Sr than the majority of Zone 2.

In the south (Zone 4), two samples from the mouth of Andoma River on the eastern side of Lake Onega and a further two on the western side yielded ^87^Sr/^86^Sr values of 0.722 and 0.723. All other data available for the Devonian areas of the EEP exhibited more typical values, staying substantially below 0.720 (Petersone-Gordina et al. 2022; Price et al. 2019). As anticipated earlier (see Geology of the Lake Onega region section), this difference is consistent with the sparse information obtainable about a narrow strip of purportedly siliciclastic terrigenous sediments on the immediate southern shore area of Lake Onega (Kolodyazhny et al. 2020; Mikuláš et al. 2013), similar to the Vendian sandstones in Zone 3. Therefore, the immediate shore area in the south is likely to show these more elevated ^87^Sr/^86^Sr values, which are not retained when leaving the shore area, where ^87^Sr/^86^Sr drops to the levels characteristic for the EEP. Ultimately, the ^87^Sr/^86^Sr obtained on the different zones did not show statistically significant differences, apart from a contrast with Zone 4 (EEP). This is common when data numbers are small and there is scatter but trends aligning with our predictions based on the local geological setting can still be observed. However, the varying degrees of overlap between the different units make it difficult to clearly assign ^87^Sr/^86^Sr values to one specific zone. Still, the biological baseline samples indicate that ^87^Sr/^86^Sr ratios above 0.724 most likely originated in the northern shore zone, specifically on the terrain of the Zaonega Fm, and values below 0.718 on the EEP. So far, values within this span cannot be securely assigned to one of the geographical zones.

In addition to samples relating to the local geological settings, there were also a small number of samples representing the Lake Onega aquatic system, consisting of modern fish and water samples from Lake Onega. The fish samples plotted substantially above the lake water ^87^Sr/^86^Sr value, but all aquatic samples fell within the span of the bedrock samples from the northern and south-south-eastern shore zones, with a minor tendency towards the northern rather than southern samples. This generally makes sense, as the largest tributaries of Lake Onega come from the north. Nevertheless, due to the size of Lake Onega and the small number of samples analysed, the possibility that there might be differences in ^87^Sr/^86^Sr values within different parts of the lake cannot be ruled out. While the samples from the area of Yuzhniy Oleniy Island and the one from Petrozavodsk Bay yielded similar ^87^Sr/^86^Sr values, it is possible that bays and arms fed by different river systems, and potentially the smaller lakes present on Zaonezhye might have different local ^87^Sr/^86^Sr signatures (e.g. Demonterova et al. 2022).

### Provenancing the burial population

Interpreting the strontium ratios acquired at YOO is complex due to the already mentioned highly diverse bedrock geology, restricted access to environmental/reference samples and higher resolution map resources, as well as the quandaries associated with HGF residential patterns. Nevertheless, our results indicate that broad assessments of population provenance are possible.

One of the goals of this study was to evaluate whether the YOO burial ground was a local phenomenon or also attracted further removed communities. Considering the measured human ^87^Sr/^86^Sr values, all of them fall within the span obtained from the baseline samples. The majority is also consistent with environmental samples sourced from areas underlain by the Zaonega Fm associated with the Paleoproterozoic basement rocks found in the areas of the lake’s northern shores and archipelago (Zone 2). Accordingly, we consider a semi-permanent residence on the northern shores of Lake Onega the most likely option, due to the following considerations: First, it is the spatially closest geological unit with a fitting ^87^Sr/^86^Sr signal, which makes it a more likely area of origin compared to more distant places with potentially similar ^87^Sr/^86^Sr values. Second, although the chronology of archaeological sites surrounding Lake Onega is subject to ongoing discussion (Filatova 2004; Tarasov 2018), multiple clusters of Mesolithic sites have been identified so far (Fig. 7). These sites include contexts interpreted as house structures suitable for year-round habitation (Filatova 2004). Considering the proposed richness of resources in and around Lake Onega, access to different ecological patches within close range specifically in the Zaonezhye area and a diet apparently mostly focused on aquatic exploitation (Schulting et al. 2022), this would allow an increasingly residential lifestyle already during the Late Mesolithic. An overall decrease in residential mobility has been proposed as a general trend for the Mesolithic (e.g. Boethius 2017; Dimitrijevic et al. 2016; Hertell and Tallavaara 2011), even though the extent of this appears to be variable (e.g. Piličiauskas et al. 2021). Third, an additional consideration would be the impact of the 8.2 ka event. As proposed by Schulting and colleagues (2022), this cold event could have led to an increased reliance on the resources of Lake Onega, compared to surrounding river and lake systems, potentially increasing the human population density in the area, and leading to territorial concerns, which could have ultimately resulted in the creation of YOO (Schulting et al. 2022). Increased territoriality and population density can in turn correlate with decreased territory size (Hamilton et al. 2007) and increased permanence of residence, in line with a semi-permanent residence of the burial population.

Finally, the material culture associated with YOO is most commonly encountered within the Lake Onega area – Filatova (2004) even considers it to be largely restricted to the shores of Lake Onega and neighbouring Lake Syamozero. In contrast, the sites further to the north and on the White Sea shores, which yielded ^87^Sr/^86^Sr values similar to Zone 2, appear to belong to a different cultural tradition (Filatova 2004; Shakhnovich 2007), further supporting the local origin of the buried individuals.

To estimate whether more confined zones of origin within the lake area could be identified, the source of the ^87^Sr/^86^Sr in the YOO individuals needs to be considered: ingested diet (Klusek 1984; Lahtinen et al. 2021). One indicator suitable for investigating this are the archaeological faunal samples from YOO, which can be assumed to have contributed to human diet. Since the majority of archaeological animals measured here are cervid remains from the burial site, we do not know where their home range was originally located. Yet, they show a very high overlap with the measured ^87^Sr/^86^Sr values of the human population (Fig. 3), which could indicate that humans and their prey largely shared the same home range and acquired their ^87^Sr/^86^Sr from the same sources of bioavailable strontium. In this case, it would be possible to compare the human ^87^Sr/^86^Sr values to the established terrestrial geological zones without further issue.

However, results of studies on the stable carbon and nitrogen isotope values of collagen clearly show the importance of freshwater aquatic resources for the subsistence of the YOO people (Eckelmann et al. in prep.; Schulting et al. 2022), and the aquatic ^87^Sr/^86^Sr values are quite a bit lower than the observed archaeological human and animal values (Fig. 3). In addition, if fish were the driver of human ^87^Sr/^86^Sr expression, further attempts at locating terrestrial habitat ranges would be in vain, since everything should theoretically be overlayed by the ^87^Sr/^86^Sr signal of Lake Onega. To clarify this, we have to consider why these people apparently show ^87^Sr/^86^Sr values that conform with terrestrial game even though their diet was supposedly predominantly comprised of aquatic resources.

Presupposing the accuracy of our aquatic baseline, two plausible scenarios could explain this apparent corundum: The first, even if we consider this unlikely, is the possibility of variance in Lake Onega ^87^Sr/^86^Sr discussed above and the chance that fish could have been mostly caught in adjacent rivers or lakes instead of Lake Onega itself. The second and more probable factor concerns the concentration of Sr in different food sources and their contributions to diet. In general, fish from Lake Onega exhibit relatively high concentrations of Sr compared to those known from most game and meat in general (Varo 1984) even if fishbones are not included in our measurements. Yet, as a rule leafy plants are considered to be the main contributor of ^87^Sr/^86^Sr in most human diets due to their high Sr concentrations (e.g. the nettle, *Urtica dioica*, has previously been measured at 140 ppm Sr in Finland (Varo 1984) compared to up to 24 ppm Sr in perch flesh from Lake Onega) (Bentley 2006; Scharlotta et al. 2018, 2022). Even with fish as the major contribution to the YOO diet, plant foods would still have played an important although less visible role from the point of view of C and N isotopes. Measurements of δ^13^C and δ^15^N on collagen are heavily biased towards the protein portion of the diet (Ambrose and Norr, 1993; Koch 2008), thus masking low protein plant consumption. Accordingly, plant contribution was likely still substantial. Edible plants available to HGFs in the Northern European boreal zone include leafy greens such as nettles, berries, roots and rhizomes, for example wild leek, and mushrooms (Bergman et al. 2004; Bishop, 2021; Kolosova et al. 2020; Vanhanen and Pesonen 2016). Leafy greens often have especially high Sr concentrations, exceeding the measured fish values (Varo 1984). To a lesser degree, this seems to be true for roots and tuber plants as well. Thus, it is likely that even smaller amounts of plant food compared to fish would have dominated the acquired strontium signal.

To this end, it needs to be remembered that the measured human bulk ^87^Sr/^86^Sr values show an averaged signal of all locations from which an individual procured their diet during the time of tooth formation (Bentley 2006). As such, the ^87^Sr/^86^Sr signal has to be considered not primarily as an indicator of residential mobility but rather as a product of the resource catchment area used, which may change or fluctuate over time, specifically in mobile or semi-mobile communities. For YOO, this – and the site’s existence without a known connected settlement site – means that it is not possible to either identify or confirm certain habitation sites or specific places as the region of origin for the buried individuals. It is, however, possible to investigate overall trends in habitat range within the predefined geological zones. First, with the exception of the two outliers, all measured values exceed the low ^87^Sr/^86^Sr values characteristic for the Phanerozoic terrains extending far south and south-east of Lake Onega (Zone 4). Therefore, the population of YOO originated, or at least spent the majority of the tooth formation period, within the area of Zones 1–3 and not on the EEP dominated south-south-eastern Zone 4. This indicates that people living on the south-south-eastern shores of the lake and its hinterland were likely not involved in the formation of YOO.

Secondly, 94% (n = 67) of all of the measured human ^87^Sr/^86^Sr values are above 0.724, which, as described above, is consistent with environmental samples sourced from the area of the Paleoproterozoic basement rocks found in the lake’s northern shores and archipelago (Zone 2). Specifically, the baseline values consistent with the Zaonega Fm (Fig. 3, black dots in Zone 2) dominating the majority of this zone and including the immediate area close to Yuzhniy Oleniy Island, (e.g. Bolshoy Klimenitsky and Zaonezhye) have the highest overlap with the bulk of the burial population. This is intriguing because one of the major settlement clusters dated to the Mesolithic at Lake Onega is situated on the northern shore of Povenetsky Bay. Since this area falls on the Tulomozero Fm, it is associated with substantially lower values than those identified for the majority of the individuals we analysed here (Fig. 3, dark grey dots in Zone 2). It is likely that only a small number of individuals with low values in our sample but still within the main range could have potentially originated there.

However, due to the overlap between the areas with lower values in the northern, hinterland and south-south-western zones (1–3), there is also the possibility that individuals with slightly lower ^87^Sr/^86^Sr values could have come from these areas. Generally though, there are no indications that any but the outliers would have likely originated from beyond the shores of Lake Onega. There are strong arguments that the northern zone, especially the Zaonega Fm dominated areas to the south of Povenetsky and west of Zaoneshye Bay are the most likely long-term habitat area for the majority of the individuals buried at YOO.

### Intragroup dynamics and mobility

The majority of measured ^87^Sr/^86^Sr values conform with an origin from within the Palaeoproterozoic Lake Onega area of the northern shores (Zone 2), specifically the Zaonega Fm, and do not indicate a high prevalence of long-distance travel or permanent relocation within the YOO burial community. Only two individuals were identified as outliers to the main sample, with an origin most likely from outside of the Precambrian areas (Zones 1–3).

Within the main sample (excluding outliers), there were no statistically significant groupings beyond the difference between the northern and southern clusters, with the former including a substantial number of burials with ^87^Sr/^86^Sr values above 0.7300 absent in the latter (Fig. 6). While this difference could be a product of chronological shifts in mobility, it is more likely that it is the result of different social subunits using different areas of the site, since so far there are no indications for a difference in radiocarbon dates between the different site parts (Hyland and Schulting pers. comm.; Schulting et al. 2022).

While the severe damage due to the mining operation makes it impossible to investigate the original layout of the site (Gurina 1956), these observed differences between the southern and northern clusters and the potential closer grouping of similar ^87^Sr/^86^Sr values in some parts of the site could cautiously indicate deliberate groupings of individuals with similar habitat ranges at the site. These could be social subunits (e.g. related to different settlement areas and/or family groups). The general presence of subgroups within the burial population is also supported by a pilot study investigating stable hydrogen isotope ratios at YOO. It indicates a tentative correlation between individuals with the highest ^87^Sr/^86^Sr values and δ^2^H values divergent from the rest of the sample, the latter possibly being related to the use of a different water source^3^ (Hyland et al. in prep.), which again might be related to familial or territorial subunits. This is in addition to previously observed spatial differences in sex and burial goods distribution (Gurina 1956; O’Shea and Zvelebil 1984; Batanina et al. in prep.).

Investigations into an association between sex and mobility patterns did not provide significant evidence for gender-based relocation patterns indicative of patri- or matrilocal residency in our analysis (see Bentley et al. 2002; Knipper et al. 2017). While both individuals identified as outliers from the main burial sample are female, the patterns of change for double-sampled individuals could tentatively indicate a higher mobility in males within the core area. Considering the limitations on temporal constraints in bulk enamel sampling and the number of available double-samples in general this suggestion is highly speculative, though. It does, however, show that further research with a sampling setup more appropriate to investigate intra-individual changes over time is necessary and should be conducted at YOO to clarify the presence or absence of gendered movement patterns.

Regardless of sex, the double-sampled individuals exhibited a variety of results ranging from a lack of change in ^87^Sr/^86^Sr values over time to the outlier individual exhibiting potentially long-distance relocation. Still, changes were observable for the majority of individuals, even though most of these remained relatively small (0.0001–0.0010) considering the complex geological background and variability in ^87^Sr/^86^Sr values of the Lake Onega area. Since both absence of change and changes of up to 0.0039 were observed in teeth whose crown formation time largely overlapped (see AlQahtani et al. 2010), this phenomenon is puzzling. One potential explanation is that we are accessing slightly different time slices due to the bulk sampling process, another might be a relatively short duration of change in the resource base for some individuals, which did not happen for others (e.g. as a result of an extended trip or season-dependent changes in food sources). Yet, the overall relatively small intra-individual difference could be indicative of general roaming and diet sourcing within one range. Everyday mobility in HGF communities is very variable but considered to rarely exceed 10 km around a habitation site, though in fish dependant societies the presence of boats can increase this radius (see Grove 2009; Kelly 2013). Due to the high variance in geology and ^87^Sr/^86^Sr at a scale often smaller than 10 km at Lake Onega, it is possible that the observed differences could originate even from movement within a daily foraging radius. In addition, the majority of analysed samples stem from early childhood or infancy (AlQahtani et al. 2010), which would put the children at too young an age to transverse larger distances by themselves (only one sample stems from an M3 with enamel forming during early adolescence). Roaming around a settlement area and independent foraging are widely attested even for young children, and the onset of this behaviour could account for the smaller value variances (Davis and Cashdan 2019; Keith, 2005; Lew-Levy et al. 2020). The larger differences would have to originate either in movement by the caretakers or through differences in food consumption leading to the observed changes.

In comparison with results from the three measured multi-person graves, there was no significant difference between intra-person changes in values and between co-buried individuals, making it probable that those buried together could have also shared the same roaming area in their youth.

While the majority of movement seems to have occurred within the vicinity of Lake Onega’s northern shores and archipelago, the two outliers to the sample indicate that while not overwhelmingly common, individuals did travel and permanently relocate over longer distances (Hodell et al. 2004; Hughes et al. 2014). Both individual 56 (5776-007, 0.7199) and especially individual 5776-47 (no grave number, 0.7147 and 0.7158) exhibit significantly lower ^87^Sr/^86^Sr values than the remaining population, suggesting that they spent time outside of what we consider the local range. In the case of individual 5776-47, the closest known areas consistently showing such low values are either on the Kola peninsula, the southern shores of the White Sea (see SI 1, Fig. S2; SI 2, Table S1) or on the EEP (e.g. in the area of Lake Ladoga but also much further south). For example, individuals from the Mesolithic-Neolithic burial sites Spiginas and Donkalnis in Lithuania also showed comparable ^87^Sr/^86^Sr values (Piličiauskas et al. 2021).

Areas further to the east cannot be excluded, however, due to the lack of comparative strontium data from the north of European Russia. This is particularly regrettable, as the closest parallels in material culture to YOO come from the sites of Peschanitsa and Popovo to the south-east of Lake Onega, in the area of the Veretye culture (Oshibkina 2008, 2016). Samples from these sites would be expected to provide similarly low ^87^Sr/^86^Sr values due to their analogous geological background. Even though these sites show the closest material parallels from secure contexts, other very similar items to YOO and human bones were recovered during the build-up of the Ladoga Canal (Inostrantseff 1882). Those were likely water carried to the find spot but might originate from a yet unknown or destroyed former burial ground – whose community might have had ^87^Sr/^86^Sr values similar to that of individual 5776-47 if we use the ^87^Sr/^86^Sr from our samples in the area of Lake Ladoga (see SI 1, Fig. S2; SI 2, Table S1) and the available literature values (Price et al. 2019) as a proxy. It is likely that the immediate hinterland of the southern Lake Onega shore beyond the terrogenic siliclastic Devonian on the EEP would also yield such values, making this area another potential and closer point of origin. Currently there are no comparative samples available for this area though and detailed geological data is difficult to access. Unfortunately, the grave context for this individual was lost at some point after initial excavation, making it impossible to provide further details based on material culture, beyond the fact that the individual grew up at some distance from YOO, likely beyond the shores of Lake Onega, and yet was buried there as an adult.

Burial 56, on the other hand, is one of the most famous graves of YOO, as the middle individual in a triple burial known for its rich grave goods, including hundreds of animal tooth-pendants and an elk-head staff (Gurina 1956). From the perspective of ^87^Sr/^86^Sr composition, it is intriguing that the individual apparently travelled during childhood. Of the two sampled teeth (mandibular P2 and mandibular I1), the one with the younger age range (I1: approx. crown formation ca. 0.5–3.5 years of age ) exhibits values that conform to the higher ^87^Sr/^86^Sr values present within the YOO sample and the northern shore zone, whereas the older tooth (P2: approx. crown formation ca. 3.5–7 years of age) shows a substantially lower value. While it is not directly attributable to one of our geological zones, it clearly came from outside of the burial community’s usual roaming area. It is therefore possible that as a child this person relocated away from Lake Onega, only to return as an adult to be buried with two other adults, who also have values different from each other but within the local range.

### Exchange, communication and travel

The results for most individuals analysed from YOO correspond with a mobility range within the Paleoproterozoic rocks most common in the northern lake zone (2). Similarly, while the double samples indicate that individuals did move, the focus of that movement stays within the range of the core distribution of the YOO sample. This fits with an increasing number of studies that point to a high degree of sedentism and low residential mobility for Late Mesolithic foraging groups primarily relying on aquatic resources, mirroring ethnographic observations (e.g. Boethius 2017; Dimitrijevic et al. 2016; Hertell and Tallavaara 2011; Schulting 2010). These indicate at least semi-permanent settlements that were often inhabited for long periods of time and survived on year-round provision of aquatic food sources by close bodies of water, with mobility mostly taking the form of logistical mobility as trips from and back to the main site (see Kelly 1983 279f.). It is very likely that this model was also practised by the communities close to YOO, as the structures at the majority of identified habitation sites include evidence of pit dwellings, which would be suitable for multi-season and potentially even year-round habitation (see Filatova 2004; Fretheim, 2017). Accordingly, the sites have been addressed in previous publications (Filatova 2004) as most likely permanent settlements. In this scenario, the YOO burial site would have served predominantly as a site of spatialized communication for the local population, apparently focused on communities in the area of Zaoneshye and the archipelago and excluding the area beyond the lake’s immediate southernmost shores, rather than as a transregional materialization of exchange networks as some previous studies suggested (O’Shea and Zvelebil 1984).

Still, while long-distance exchange apparently was not the driver behind the establishment of the YOO site, the connections that did exist between the Lake Onega community and other Mesolithic communities are highlighted by a number of grave finds and the two individuals with ^87^Sr/^86^Sr values outside the range of the main sample. As with the outlier individuals, the finds of non-local origin also point to the south and east, rather than the north. They include 152 flint objects, for which the closest sources would be located in the coal seams that stretch from shortly beyond the south-eastern tip of Lake Onega east and northwards all the way to the White Sea (Gurina 1956; Kosorukova, 2017; O’Shea and Zvelebil 1984), as well as animal-bone and tooth artefacts made of species adapted to more temperate habitats. These faunal artefacts include pendants identified via ZooMS as bovid, which in this context must mean either bison (*Bison bonasus*) or aurochs (*Bos primigenus*) (Mannermaa et al. in prep.). Likewise, boar-tooth pendants identified in two burials should likely also be considered as imports (Mannermaa et al. 2019, in prep.). Even though a small local presence of these animals cannot be completely excluded, no other finds of these species are known from this area in the Late Mesolithic. In addition, the ecology of wild bovids and boars indicates that the cold climate specifically of the 8.2 ka event would not be favourable to their presence (Ukkonen et al. 2015). Thus, it appears more likely that these finds were brought from outside the Lake Onega region, probably from the south. This is corroborated in the case of one boar tooth by its ^87^Sr/^86^Sr signature of 0.7198, which, considering potential warmer areas of origin, likely points to the EEP, even though the associated human individual shows local values.

Hence, materials seem to have travelled towards Lake Onega even over great distances, and the presence of the two outlier individuals provides a high likelihood that these exchanges did not solely rely on the passing on of goods through various hands but also involved long-distance travel of people. This observation is not in contradiction with what has previously been stated about the most likely relatively local origin of the people buried at YOO. Their everyday lives would have consisted of mostly local mobility, with additional more extensive provisioning trips. Long journeys and permanent relocation are only rarely visible in the strontium isotope values and may have been undertaken only by select individuals (e.g. for exchange or marital purposes).

Considering the known Mesolithic burial sites, the closest parallels to YOO in terms of material culture are the Popovo and Peschanitsa burial sites, close to Lake Lacha to the south-east (Oshibkina 2008, 2016). This is also consistent with ideas about the origin of the Onega culture and its early connections to Butovo and Veretye (Filatova 2004; Oshibkina 2008; Tarasov 2018). Since there are currently no available ^87^Sr/^86^Sr values for these sites, a direct comparison is not possible, but as they are also situated on the EEP, it is likely that relatively low ^87^Sr/^86^Sr values consistent with the outlier values measured here could be expected. Direct material connections to the Mesolithic sites of the Baltic area (e.g. Zvejnieki, Skateholm) are less evident (e.g. Larsson 1988; Macāne 2022). There are cases of ^87^Sr/^86^Sr outliers in both individuals (e.g. Spiginas in Lithuania; Piličiauskas et al. 2021) and artefacts (e.g. Skateholm, Sweden; Larsson and Price 2022) at these sites though, which yielded ^87^Sr/^86^Sr values congruent with the high ^87^Sr/^86^Sr values of the FSS and likely were not of local origin. These further seem to indicate that at least semi-regular movement to and from the south appears to have been an occasional yet constant phenomenon.

## Conclusions

The first investigation of ^87^Sr/^86^Sr values from the Mesolithic site of YOO suggests that this famous and large cemetery served as a burial site primarily for local communities for the northern area of Lake Onega, rather than as a centre for long-distance connections. This is in concert with previous assessments of a more localised cultural focus at the end of the Mesolithic (Filatova 2004), compared to the previous phase of population expansion towards the north after the end of the last glaciation (e.g. Riede and Tallaavaara 2014; Tarasov 2018), as well as during the subsequent pottery Neolithic and Eneolithic. The latter especially show the activation of intense long-distance exchange, one of the most striking examples of which is the export of metatuff axes and adzes from the shores of Lake Onega (Tarasov and Nordqvist 2022).

While it was not possible to match specific individuals from the cemetery to site clusters on the shores of Lake Onega, the combined presence of spatial variability in ^87^Sr/^86^Sr values at the site and other archaeological proxies indicate the presence of subgroups that are also associated with differing geological markers. The presence of these subgroups, combined with the overall coherence of ^87^Sr/^86^Sr values and material culture, support the idea that YOO may have played an important role as a site of communication and negotiation for the communities sharing the resources of Lake Onega and its surroundings, specifically those living on the north-north-western shores and the archipelago (e.g. Filatova 2004; Jacobs 1995; O’Shea and Zvelebil 1984; Schulting et al. 2022). In terms of movement patterns, the double-sampled individuals showed varying degrees of mobility, yet predominantly stayed within the common YOO range, which supports the notion of a relatively high degree of residential stability, fitting overall trends in the Late Mesolithic, specifically in societies relying on aquatic resources. Long-distance contacts are nevertheless present in the form of a number of artefacts indicating a non-local origin, as well as two individuals identified as having spent time outside of the wider Lake Onega area. One of these individuals (burial 56) apparently left the core range of the sample during childhood, only to later return and be buried at YOO in one of the richest furnished graves of the burial site. This puts the Lake Onega community within the wider Mesolithic communication network and fits well with previous results, showing wide-reaching movement between Mesolithic communities. Even if there seems to be a reduction in material exchange between the Onega area and other regions in the Late Mesolithic (see Gerasimov et al. 2010; Kriiska et al. 2016), this is not reflected in a complete cessation of individual travel.

Our study has provided a glimpse of the potential information that can be gained from long-known sites through the application of modern methods. Accordingly, it would be desirable to continue this investigation in the future. Expanding and increasing data on the local and regional strontium baseline around Lake Onega and including Lake Syamozero, where other sites attributed to the Onega culture are situated, has the potential to unravel the connections between ^87^Sr/^86^Sr clusters at the burial site and site clusters in the Lake Onega area. Ideally, this would also include more archaeological samples and radiocarbon dates (e.g. on faunal remains from settlement and camp sites). Similarly, more precise interindividual analysis via laser ablation (e.g. Boethius et al. 2022a, b) or multi-sampling would contribute to increased understandings of both the impact and extent of intra-range movement, especially if comparative values from further sites (e.g. to the east) were available as well. Lastly, investigations into markers of biological relatedness combined with spatial markers could also clarify the role of YOO as a locale of communication and consolidation of communal bonds.

## Supplementary information

Below is the link to the electronic supplementary material

ESM 1 (DOCX 2.91 MB)

ESM 2 (XLSX 44.6 KB)

## Data Availability

Data is provided within the manuscript and supplementary information files.

## Footnotes

1 Оленеостровский могильник in the original publication, previously also referred to in English transcriptions as Oleni’i Island, Oleniostrov and Oleneostrovskiy cemetery.

2 N. Gurina estimates the original size of the site as up to 500 graves, based on the size of the quarry mine, but does not provide further arguments for this number (Gurina, 1956).

3 Or considering the size of Lake Onega maybe a different bay or river delta.
