# Supplementary material for: Mobility and community at Mesolithic Lake Onega, Karelia, north-west Russia: insights from strontium isotope analysis
Source: Archaeol Anthropol Sci. 2024 Dec 30;17(1):17. doi: 10.1007/s12520-024-02129-8 (PMC11685259; doi:10.1007/s12520-024-02129-8)
Supplement: Supplementary file 1 — (DOCX 2.91 MB) [file 12520_2024_2129_MOESM1_ESM.docx]

**Mobility and Community at Mesolithic Lake Onega, Karelia, northwest Russia – Insights from strontium isotope analysis**

**Supplementary Information 1**

Rebekka Eckelmann, Laura Arppe, Alexey Tarasov, Łukasz Pospieszny, Lukáš Ackerman, Volker Heyd, Dmitry Gerasimov, Vyacheslav Moiseyev, Vanessa Fairbanks, Corrie Hyland, Kristiina Mannermaa

Corresponding Author: Rebekka Eckelmann

Contact: [rebekka.eckelmann@helsinki.fi](mailto:rebekka.eckelmann@helsinki.fi)

Journal: Archaeological and Anthropological Sciences

| 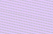 | Sandstone, conglomerate, siltstone, shale (Vendian to Lower Cambrian) |
| --- | --- |
| 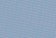 | Red sandstone and mudstone, conglomerate, metasandstone, quartzite, phyllite, volcanic and metavolcanic rock |
| 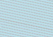 | Limestone, marl, coal, oil shale, shale, siltstone, sandstone, conglomerate (Middle Cambrian to Permian) |
| 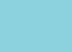 | Metagreywacke, metasiltstone, metasandstone, mica schist, graphite- and/or sulphide-bearing schist, paragneiss, amphibolite intercalations (c. 1.95-1.87 Ga and possibly older) |
| 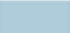 | Black schist, carbonaceous quartzite, siltstone, shungitic rocks, dolostone, limestone, basalt, andesitic basalt, picrobasalt/dolerite |
| 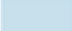 | Dolostone, stromatolitic dolostone, arkosic sandstone, quartzite, siltstone, limestone, basalt |
| 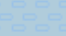 | Mica schist, conglomerate, gritstone, diamictite, arkosic sandstone, quartzite, tuffite |
| 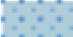 | Quartzite, mica schist, mica gneiss, conglomerate |
| 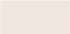 | Tonalite-trondhjemite-granodiorite gneiss, quartzo-feldspathic gneiss, enderbite, migmatitic gneiss, with mafic and felsic enclaves (c. 3.20-2.65 Ga and possibly older) |
| 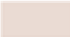 | Diorite, tonalite, granodiorite, trondhjemite, enderbite, charnockite (c. 3.00-2.74 Ga) |
| 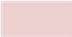 | Granite, pegmatite (c. 2.70-2.65 Ga) |
| 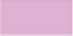 | Granite, quartz syenite, quartz monzonite, monzonite, charnockite |
| 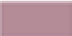 | Layered intrusion: gabbro, gabbro-norite, anorthosite, dunite, peridotite, pyroxenite |
| 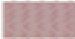 | Ferrodolerite, monzodiorite |
| 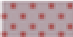 | Gabbro, monzodiorite, syenite, granodiorite (c. 2.74-2.65 Ga) |
| 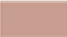 | Dolerite, gabbro, metagabbro (c. 1.77 Ga) |
| 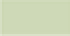 | Komatiite, basalt, andesite, dacite, rhyolite |
| 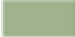 | Basalt, high-Mg basalt, high-Mg andesite, dacite, komatiitic basalt/dolerite |
| 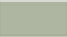 | Tholeiitic, komatiitic and Fe-rich tholeiitic basalt, peridotite, gabbro, dacite, rhyolite, conglomerate |
| 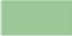 | Tholeiitic basalt, subordinate quartzite and conglomerate |
| 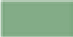 | Tholeiitic basalt, ferropicrite, picrite, peridotite, pyroxenite, gabbro, wehrlite/dolerite |

**Fig. S1** Full version of the legend presented within Fig. 2a showing the detailed bedrock geology of the Lake Onega region (Koistinen et al. 2001, reproduced with the permission of The Federal Agency of Use of Mineral Resources of the Ministry of Natural Resources of the Russian Federation (MNRRF) from https://gtkdata.gtk.fi/fmd/ 9.11.2023)


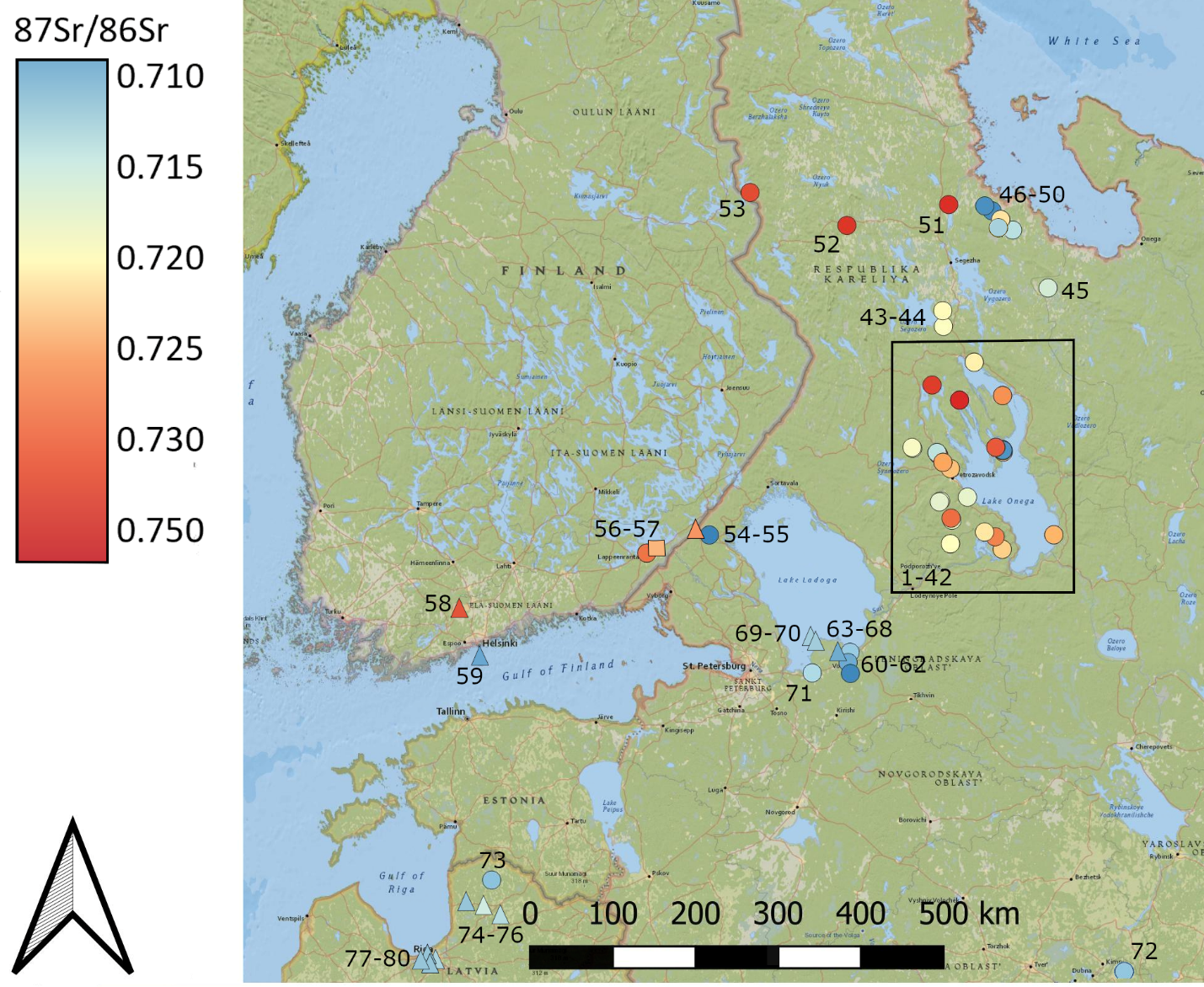


**Fig. S2** Map with ^87^Sr/^86^Sr values of bioavailable baseline samples (color-coded; circles represent plants, squares water and triangles faunal samples; for numbered list see SI 2 Tab. S1). The black box indicates the primary sampling area detailed in Fig. 7

**Fig. S3** Sr concentration values plotted against ^87^Sr/^86^Sr. The group of faunal samples with Sr concentration values above 1000 ppm were excluded from analysis
